# Identification of *Streptococcus thermophilus* Genes Specifically Expressed under Simulated Human Digestive Conditions Using R-IVET Technology

**DOI:** 10.3390/microorganisms9061113

**Published:** 2021-05-21

**Authors:** Ophélie Uriot, Mounira Kebouchi, Emilie Lorson, Wessam Galia, Sylvain Denis, Sandrine Chalancon, Zeeshan Hafeez, Emeline Roux, Magali Genay, Stéphanie Blanquet-Diot, Annie Dary-Mourot

**Affiliations:** 1 EA 7488 Calbinotox Composés Alimentaires Biofonctionnalités & Risque Neurotoxique, Université de Lorraine, 54506 Vandoeuvre-lès-Nancy, France; ophelie.uriot@uca.fr (O.U.); mounira.kebouchi@hotmail.fr (M.K.); emilie.lorson@univ-lorraine.fr (E.L.); wessam.galia@vetagro-sup.fr (W.G.); zeeshan.hafeez@univ-lorraine.fr (Z.H.); emeline.roux@univ-lorraine.fr (E.R.); magali.genay@univ-lorraine.fr (M.G.); 2 UMR 454 MEDIS Microbiology, Digestive Environment and Health, Université Clermont Auvergne, INRAe, 63000 Clermont-Ferrand, France; sylvain.denis@uca.fr (S.D.); sandrine.chalancon@uca.fr (S.C.); stephanie.blanquet@uca.fr (S.B.-D.); 3 UMR 5557 Microbial Ecology, Research Group on Bacterial Opportunistic Pathogens and Environment, CNRS, VetAgro Sup, 69280 Marcy L’Etoile, France; 4 INRIA/IRISA, GenScale Bioinformatics Team, 35042 Rennes, France

**Keywords:** *S. thermophilus*, R-IVET, TIM-1 system, intestinal microbiota, adhesion

## Abstract

Despite promising health effects, the probiotic status of *Streptococcus thermophilus,* a lactic acid bacterium widely used in dairy industry, requires further documentation of its physiological status during human gastrointestinal passage. This study aimed to apply recombinant-based in vivo technology (R-IVET) to identify genes triggered in a *S. thermophilus* LMD-9 reference strain under simulated digestive conditions. First, the R-IVET chromosomal cassette and plasmid genomic library were designed to positively select activated genes. Second, recombinant clones were introduced into complementary models mimicking the human gut, the Netherlands Organization for Applied Scientific Research (TNO) gastrointestinal model imitating the human stomach and small intestine, the Caco-2 TC7 cell line as a model of intestinal epithelium, and anaerobic batch cultures of human feces as a colon model. All inserts of activated clones displayed a promoter activity that differed from one digestive condition to another. Our results also showed that *S. thermophilus* adapted its metabolism to stressful conditions found in the gastric and colonic competitive environment and modified its surface proteins during adhesion to Caco-2 TC7 cells. Activated genes were investigated in a collection of *S. thermophilus* strains showing various resistance levels to gastrointestinal stresses, a first stage in the identification of gut resistance markers and a key step in probiotic selection.

## 1. Introduction

The lactic acid bacterium *Streptococcus thermophilus* is widely used as a starter for yogurt and cheese production, mainly for its ability to produce lactic acid and secondary fermentation products with aromatic and textural properties [1]. It has a very long history of use in dairy industry without any identified health problem. Furthermore, virulence-related genes are absent from its genome [2]. Hence, it has been assigned the generally recognized as safe (GRAS) and qualified presumption of safety (QPS) status by the American Food and Drug Administration and European Food Safety Authority (EFSA), respectively. In spite of its very large human consumption and the health claim attributed by EFSA to live yogurt cultures (*S. thermophilus* and *Lactobacillus delbrueckii*) to improve lactose digestion [3], the probiotic status of *S. thermophilus* strains is still poorly explored [4,5].

Probiotics are defined as “live micro-organisms that, when administered in adequate amounts, confer a health benefit on the host” [5,6]. The main criteria to select probiotics include (i) resistance to stresses encountered in the human gastrointestinal (GI) tract such as acidic pH and bile salts, and (ii) beneficial impacts on host health. Few studies have already shown that *S. thermophilus* can survive passage through the human gut [7,8,9,10]. Nevertheless, it must be underlined that bacterial counts were mostly performed in the feces of human volunteers, not along the entire GI tract, and after consumption of yogurts containing both *S. thermophilus* and *L. delbrueckii*, which can affect survival of individual strains. Although survival of probiotics in the human gut involves resistance to main GI stresses, capacity to adhere to intestinal epithelial cells may also contribute to their persistence in the GI tract by promoting a transient intestinal colonization [11,12]. In vitro studies have shown that few strains of *S. thermophilus*, especially the LMD-9 reference strain, are able to adhere to several types of human intestinal epithelial cell lines [13,14,15,16]. Regarding health effects, in vitro studies and in vivo experiments in rodents have shown that *S. thermophilus* possesses interesting properties (in addition to lactose digestion improvement) such as prevention of chronic gastritis and antimicrobial or antioxidant activities, as reviewed by Uriot et al. [17]. Interestingly, it was shown that all these properties were largely strain-dependent [15,18,19]. Consequently, additional information on the survival and metabolic status of this bacterium in the human digestive environment is compulsory before assigning any probiotic allegation.

Several global approaches can be employed to address the metabolic status of *S. thermophilus* in the digestive tract, such as DNA microrarray, RNA-sequencing, or proteomics, but few studies have been done. Only two proteomics investigations have been performed to attempt to establish the physiological status of *S. thermophilus* during passage through the GI tract. Results have highlighted the importance of the glycolysis pathway [20,21]. Nevertheless, these studies were carried out in gnotobiotic rats, a model that is far from the complex human GI tract physiology. Another strategy based on analysis of the bacterial transcriptome is the recombinase-based in vivo expression technology (R-IVET). R-IVET is a promoter-trapping technology that consists of two elements: (i) a plasmid containing a promoterless recombinase gene (often the gene *cre*) upstream of which genomic DNA fragments are inserted, and (ii) a chromosomal cassette with at least an antibiotic resistance gene flanked by recognition sites (often the *loxP* sequences, recognized by the recombinase Cre) of plasmid-encoded recombinase. Therefore, if a genomic DNA fragment inserted upstream of the recombinase gene displays promoter activity (activated R-IVET recombinant clone), the recombinase is expressed and the antibiotic resistance gene located in the chromosomal cassette is excised [22,23]. The R-IVET approach has already been developed in different bacteria, such as *Bifidobacterium longum*, *Lactobacillus plantarum*, *Vibrio cholerae*, and *S. thermophilus* [23,24,25,26,27]. Up to now, this technique was used to follow gene expression in complex digestive media, but mainly when using mice models [24,25,26,27]; alternatively, in a unique previous study by our team, it was used to assess the behavior of the *S. thermophilus* LMD-9 reference strain under simulated human gastric conditions [18]. However, this technology is often limited by its screening phase. Most of the time, a single resistance gene inside the chromosomal cassette results in a cumbersome selection of activated clones, leading to a heavy and time-consuming negative screening.

The aim of this work was (i) to optimize the R-IVET tool for *S. thermophilus* LMD-9 by designing and validating a chromosomal cassette that allows a positive screening of activated recombinant R-IVET clones, and (ii) to use the R-IVET approach to identify which *S. thermophilus* LMD-9 genes are specifically activated during transit through the entire simulated human GI tract. In an original and global approach, we used, for the first time, three complementary in vitro human gut models: (i) the dynamic multicompartmental Netherlands Organization for Applied Scientific Research (TNO) gastrointestinal model (TIM-1), which is currently the most complete simulator of physicochemical conditions found in the human stomach and small intestine [28,29,30], (ii) the Caco-2 TC7 cell line which exhibits a morphological and functional enterocyte phenotype closed to that found in humans (for a review, see [31]) as a model of bacterial adhesion to human intestinal epithelial cells, and (iii) anaerobic batch cultures of human feces as a simplified model of the human colon.

## 2. Materials and Methods

### 2.1. Bacterial Strains, Culture, and Transformation Conditions

Bacterial strains and plasmids used in the present study are listed in Table 1. *S. thermophilus* LMD-9 from the American Type Culture Collection (Manassas, VA, USA) was used to implement the R-IVET system. *S. thermophilus* strains were stored at −80 °C in reconstituted skim milk (10%, *w*/*v*). They were grown at 42 °C under anaerobic conditions (AnaeroGen, Oxoid, Basingstoke, UK) in M17 medium [32] supplemented with 2% (*w*/*v*) lactose (LM17) or in 10% (*w*/*v*) reconstituted milk (powdered semi-skimmed milk, fast dissolution, Régilait). Unless otherwise specified, antibiotics (Sigma, Saint Quentin Fallavier, France) were added at the following concentrations: spectinomycin 300 µg/mL, streptomycin 20 µg/mL, kanamycin 1 mg/mL, or erythromycin 5 µg/mL. *S. thermophilus* naturally competent cells were prepared in Chemically Defined Medium (CDM) as previously described [33] and transformed according to Junjua et al. [23]. For long-term storage at −80 °C, competent cells were centrifuged, resuspended in 1/10 volume of the initial culture supplemented with glycerol (14%), and frozen in liquid nitrogen. As *S. thermophilus* develops in the form of coccus chains, samples were systematically vigorously vortexed for 3 min to break the chains before spreading on solid medium. This step was to ensure that each colony observed after incubation at 42 °C probably came from a single cell.

*E. coli* TOP10 (Invitrogen, Breda, The Netherlands) was used as an intermediate cloning host and was grown in aerobic conditions at 37 °C in Luria–Bertani (LB) medium under shaking at 240 rpm, supplemented or not with erythromycin 300 µg/mL. Chemical transformation of this strain was performed according to manufacturer’s instructions.

### 2.2. DNA Extraction, Digestion, and PCR Amplification

*S. thermophilus* genomic DNA was extracted as previously described by Fischer et al. [36], and restriction digestions were performed following the manufacturer’s instructions. Polymerase chain reactions (PCR) were carried out in a Mastercycler pro thermocycler (Eppendorf, Hambourg, Germany). Oligonucleotides used as primers were purchased from Eurogentec (Seraing, Belgium), and their sequences are described in Table S1 (Supplementary Materials). Classic and overlapping PCRs were performed using enzymes and conditions previously described by Junjua et al. [23]. DNA fragments were separated by electrophoresis in 1% agarose gels using 0.5× Tris-Borate EDTA buffer at 100 V [37]. Molecular weight marker 1 kb and 100 bp DNA ladders from Fermentas were used. Gels were stained with ethidium bromide and imaged using a GelDoc-It Imaging System (Bio-Rad, Marne-la-Coquette, France).

### 2.3. Construction of R-IVET Chromosomal Cassette and S. thermophilus Mutant STUL5003

**Figure 1:**
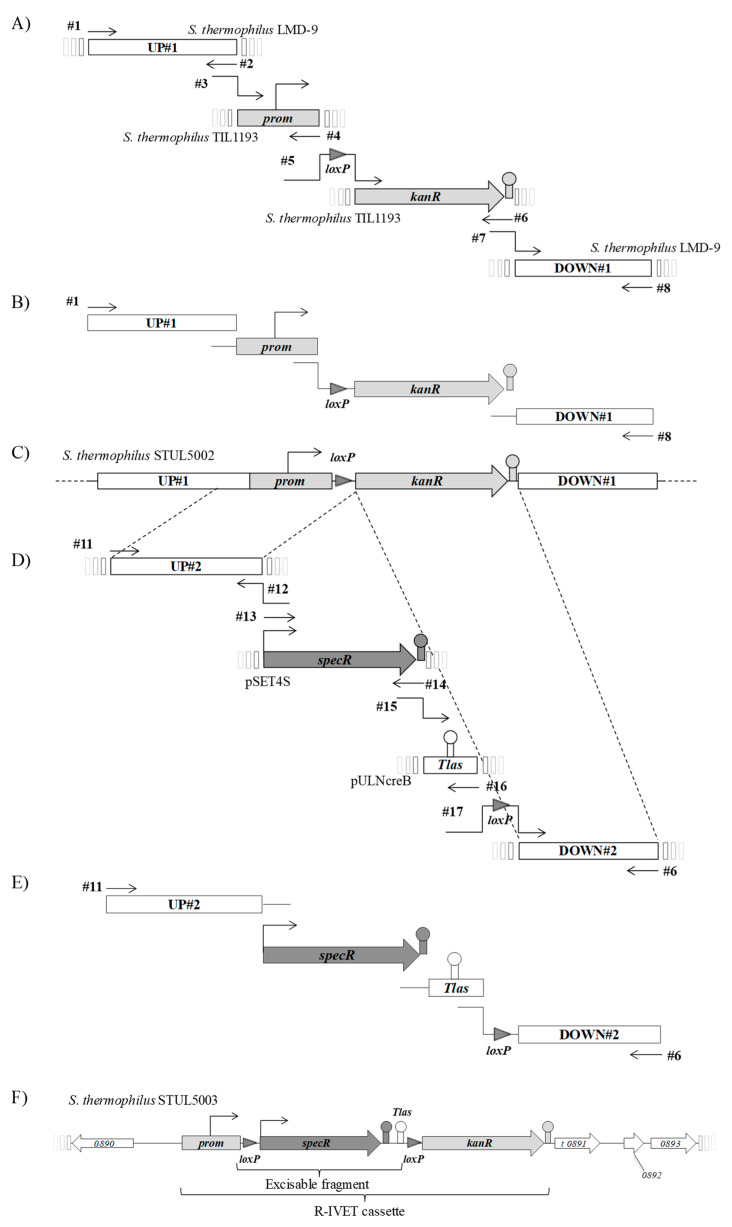
Construction of the chromosomal R-IVET cassette. The R-IVET chromosomal construction was cloned into the locus STER 0891 (*t0891*) of *S. thermophilus* LMD-9 resulting in strain STUL5003. This strain was constructed in two steps. The first step (1A to 1C) corresponds to the construction of STUL5002 mutant: PCR amplification of individual fragments (**A**), overlapping PCR (**B**), and resulting construction on the chromosome of STUL5002 mutant (**C**). The second step (1D to 1F) illustrates the construction of STUL5003 mutant: PCR amplification of individual fragments (**D**), overlapping PCR (**E**), and resulting chromosomal construction of STUL5003 mutant (**F**). Numerical values indicated in italics in genes (**F**) correspond to locus tags as described in the annotated sequence of LMD-9 chromosome (NC_008532_1). Oligonucleoti0des used as amplification primers are indicated as black arrows and are labeled with their # numbers (see Table S1, Supplementary Materials). UP and DOWN regions correspond to upstream and downstream sequences flanking the fragments to be cloned, respectively. The *specR, kanR*, and *prom* fragments correspond to genes encoding a spectinomycin adenyltransferase conferring resistance to spectinomycin and streptomycin, a 3′5″-aminoglycoside phosphotransferase of type III conferring resistance to kanamycin, and the promoter region of *kanR* gene, respectively. The gray triangle corresponds to the *loxP* sequence that is recognized by the specific site recombinase Cre.

The strategy used to construct the *S. thermophilus* STUL5003 mutant strain containing the chromosomal *prom–loxP–specR–Tlas–loxP–kanR* cassette is presented in Figure 1. In a first series of PCR (Figure 1A–C), the intermediate strain STUL5002 was built from *S. thermophilus* LMD-9. This mutant strain contained a chromosomal cassette consisting of the *kanR* gene, encoding a 3′5′-aminoglycoside phosphotransferase of type III (*aphA3*), separated from its own promoter by a *loxP* site (Figure 1C). The presence of *loxP* did not preclude the expression of the *kanR* gene. To obtain the cassette, a series of PCRs was carried out as follows: amplification of the UP#1 and DOWN#1 fragments from genomic DNA of *S. thermophilus* LMD-9, and of the *prom* (promoter region of the *kanR* gene) and *loxP–kanR* fragments (Figure 1A) from genomic DNA of *S. thermophilus* TIL1193 (Table 1) [33]. The four fragments UP#1, *prom*, *loxP–kanR*, and DOWN#1 were amplified individually using primer couples #1#2, #3#4, #5#6, and #7#8, respectively (Figure 1A). Then, an overlapping PCR was carried out using primer couples #1#8 and the four fragments UP#1, *prom*, *loxP–kanR*, and DOWN#1 mixed together in equimolar concentrations (Figure 1B). About 30 ng of the resulting amplified fragments were used for transformation of LMD-9 competent cells. A kanamycin-resistant clone was selected and confirmed to have the *prom–loxP–kanR* fragment at the STER_0891 locus by colony PCR using the primer couple #9#10 (Figure 1C). The resulting mutant strain was named STUL5002. The *prom–loxP–kanR* fragment and chromosomal junctional regions were sequenced to check that no mutation occurred during this mutant construction.

In a second series of PCR (Figure 1D–F), strain STUL5003 containing a *prom–loxP–specR–Tlas–loxP–kanR* chromosomal cassette was built. The four fragments UP#2, *specR*, *Tlas*, and *loxP*–DOWN#2 were individually amplified using primer couples #11#12, #13#14, #15#16 and #17#6, respectively (Figure 1D). Genomic DNA of strain STUL5002 was used as template to amplify the UP#2 and *loxP–*DOWN#2 fragments. The terminator *Tlas* fragment was amplified from the pULNcreB plasmid [23]. The plasmid pSET4S (Table 1, [35]) was used to amplify the *specR* gene encoding a spectinomycin adenyltransferase. Once obtained, fragments UP#2, *specR*, *Tlas*, and *loxP–*DOWN#2 were mixed together in equimolar concentrations, and an overlapping PCR was carried out using primer couple #11#6 (Figure 1E). About 30 ng of the resulting amplified fragments were used for transformation of STUL5002 competent cells (Figure 1F). Among the spectinomycin-resistant transformants, three were randomly selected, and the construction was sequenced in each to confirm the presence of the expected chromosomal cassette. Hence, one of them was chosen and named STUL5003. This strain displayed a Spec^R^Kan^S^ phenotype as expected.

### 2.4. R-IVET Genomic Library Construction

Digestion with *Alu*I and *Sma*I restriction enzymes, ligation with the T_4_DNA ligase, and dephosphorylation using calf intestinal alkaline phosphatase were performed according to the supplier’s recommendations (New England Biolabs, Leiden, The Netherlands). The R-IVET library was constructed in the STUL5003 strain. The genomic DNA of *S. thermophilus* LMD-9 was partially digested with *Alu*I, and restriction fragments were ligated with the pULNcreB plasmid, which was previously linearized by *Sma*I and dephosphorylated. DNA digestions were checked by agarose gel electrophoresis and were purified using the High Pure DNA purification Kit (Roche Molecular Biochemicals, Mannheim, Germany) according to the manufacturer’s recommendations. Chemically competent cells of *E. coli* TOP10 were transformed with the ligated plasmids. All resulting clones were pooled and their plasmid DNAs extracted using a Miniprep Kit (Fermentas, Villebon sur Yvette, France). Plasmid DNAs were then introduced by natural transformation into *S. thermophilus* STUL5003. The resulting *S. thermophilus* clones were selected on LM17 supplemented with streptomycin, pooled, resuspended into LM17 supplemented with 11.6% glycerol, and stored in aliquots at −80 °C to constitute the *S. thermophilus* R-IVET genomic library.

### 2.5. Optimization of the Counterselection Condition of Activated R-IVET Clones

To efficiently eliminate Spec^S^Kan^R^ clones obtained during the R-IVET library growth before its introduction into one of the gut models used in this work, two different antibiotic combinations were tested: spectinomycin 300 µg/mL or a mix of spectinomycin 300 µg/mL and streptomycin 20 µg/mL. Thus, after growth in milk (medium used for TIM-1 and fecal batch culture models) or in LM17 (Caco-2 TC7 model), cells of the R-IVET genomic library were exposed for 3 h, 7 h, or 15 h to these antibiotics. Counterselection effectiveness of the Spec^S^Kan^R^ clones was then evaluated by plating appropriate dilutions of each sample on LM17 agar supplemented with erythromycin (LM17-ery), a mix of spectinomycin and streptomycin (LM17-spec/strep), or a mix of kanamycin and erythromycin (LM17-kan/ery). As the *specR* gene confers resistance to both spectinomycin and streptomycin, these antibiotics were used alone or together. After 3 h exposure to spectinomycin 300 μg/mL or a mix of spectinomycin 300 μg/mL and streptomycin 20 μg/mL, 0.15% and 0.03% of colonies plated onto LM17-ery were also present on LM17-kan/ery, respectively. The results appeared slightly better since 0.015% of colonies plated onto LM17-ery were also growing on LM17-kan/ery after 7 h exposure to the mix of spectinomycin 300 µg/mL and streptomycin 20 µg/mL vs. 0.08% when using spectinomycin 300 µg/mL. Similar results were obtained after 15 h exposure. Moreover, for each condition of counterselection tested, 10 clones randomly selected from LM17-kan/ery plates, deletion of *loxP*–*specR* fragment from the chromosomal cassette, and presence of an insert in pULNcreB plasmid were confirmed. Therefore, it appeared that counterselection using a mix of spectinomycin/streptomycin was the most efficient. On the basis of these results and for further easy handling, it was decided to grow the R-IVET genomic library overnight in milk (TIM-1 and batch cultures) or in LM17 (adhesion) with this mix of antibiotics.

### 2.6. In Vitro Digestions in the TIM-1 System

**Figure 2:**
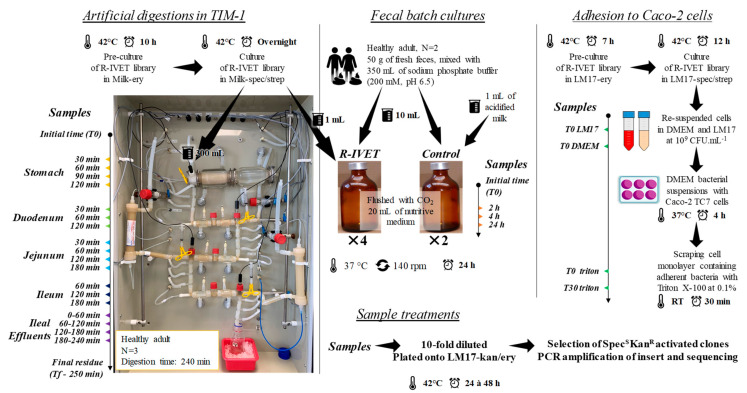
Overview of R-IVET experiments in the three complementary human gut models.

The TIM-1 system is composed of four successive compartments, namely, the stomach, duodenum, jejunum, and ileum. This computer-controlled dynamic in vitro model accurately reproduces the main physicochemical parameters found in the stomach and small intestine in vivo (no microbiota): body temperature, kinetics of gastric and intestinal pH, gastric, pancreatic, and liver digestive secretions, chyme transit and mixing, and passive absorption of nutrients and water (Figure 2). TIM-1 was washed with detergent and sterilized by steaming at 105 °C for 35 min before each experiment to avoid any microbial contamination. In the present study, the model was programmed to reproduce digestion of milk by a healthy human adult (Table 2).

R-IVET library clones were precultured for 10 h in milk supplemented with erythromycin. Then, the library was cultured overnight in 300 mL of milk supplemented with spectinomycin and streptomycin. Before introduction into the TIM-1 stomach, the fermented milk was homogenized by vortexing at room temperature for 5 min. Samples (1 mL) were collected from the initial fermented milk (T0) and at several time points during in vitro digestion in the stomach (30, 60, 90, and 120 min), duodenum (30, 60, and 120 min), jejunum (30, 60, 120, and 180 min), and ileum (60, 120, and 180 min). Ileal effluents were also kept on ice and collected as pools of periods covering 0–60, 60–120, 120–180, and 180–240 min. At the end of digestion, the gastrointestinal residue was recovered. Samples were 10-fold diluted and appropriate dilutions were plated on LM17 agar (viable count) and on LM17 agar supplemented with a mix of kanamycin and erythromycin (selection of activated Spec^S^Kan^R^ clones). Three independent experiments were performed in the TIM-1 system.

### 2.7. Fecal Batch Cultures

Fresh feces from healthy human volunteers were used to prepare the bacterial inoculum for batch cultures. Stools (~50 g) were mixed with 350 mL of sodium phosphate buffer (200 mM, pH 6.5) and filtered through a double layer of gauze under strictly anaerobic conditions in a vinyl anaerobic chamber (Coy, Grass Lake, MI, USA). Ten milliliters of the fecal suspension was rapidly transferred to 50 mL crimped vials, flushed with CO_2_, and filled with 20 mL of nutritive medium (Figure 2). The nutritive medium contained various carbohydrate, protein, lipid, mineral, and vitamin sources, as previously described by Thévenot et al. [38]. For each fecal sample, six vials were prepared and sealed: two control vials with 1 mL of acidified milk and four vials containing 1 mL of an R-IVET genomic library culture. The vials were incubated for 24 h (37 °C, 140 rpm), and samples (1 mL) were collected at 0, 2, 4, and 24 h. Appropriate dilutions were plated on LM17 agar supplemented with a mix of spectinomycin and streptomycin (viable count) and on the same medium supplemented with a mix of kanamycin and erythromycin (selection of Spec^S^Kan^R^ activated clones and plasmid presence through erythromycin resistance). The experiments were performed in duplicate with the feces of two adult volunteers, a male and a female.

### 2.8. In Vitro Adhesion to Caco-2 Cultures

The R-IVET library was precultured overnight in LM17 supplemented with erythromycin and cultured for 12 h in LM17 supplemented with spectinomycin and streptomycin. The Caco-2 TC7 cell line was used for adhesion studies, and cell culture experiments were performed as previously described by Kebouchi et al. [16] with some modifications. Briefly, bacterial cells from 12 h cultures were pelleted, washed, and resuspended at a final concentration of 10^9^ CFU/mL (OD 650 nm of about 12) in Dulbecco’s modified Eagle’s Minimal Essential Medium (DMEM) with 4.5 g/L glucose (DMEM Glutamax, Fisher Scientific) or in LM17, used as a control. Subsequently, samples were taken separately from the two bacterial suspensions corresponding to T0 LM17 and T0 DMEM samples (Figure 2). Afterward, the bacterial suspension in DMEM was added to a confluent Caco-2 TC7 cell monolayer at a final concentration of 10^9^ CFU/mL to reach a bacterial-cell-to-epithelial-cell ratio of 1000:1. Bacterial cells were co-incubated with the cell monolayer for 4 h at 37 °C in a humidified atmosphere with 10% CO_2_ and processed further according to Kebouchi et al. [16] to recover cells. All cells (adherent bacteria and eukaryotic cells) recovered from two inserts were pooled and then incubated 30 min at room temperature. Samples were taken separately after recovering from triton treatment of bacterial suspension (T0 triton) and after 30 min incubation (T30 triton). Serial dilutions of all samples (T0 LM17, T0 DMEM, T0 triton, and T30 triton) were performed, and appropriate dilutions were plated onto LM17 agar supplemented with either a mix of spectinomycin and streptomycin or a mix of kanamycin and erythromycin (selection of activated Spec^S^Kan^R^ clones having a recombinant plasmid). Plates were then incubated at 42 °C for 48 h. Three independent R-IVET adhesion experiments were conducted with Caco-2 TC7 cells.

### 2.9. Sequence Analyses

In the three gut models, excision of the chromosomal cassette was firstly checked in Spec^S^Kan^R^ clones (qualified as an activated R-IVET clone) by PCR using the primer pair #22 and #29 (Table S1, Supplementary Materials). Detection of a 628 bp amplicon confirmed that the *loxP-specR* fragment of the R-IVET cassette was deleted because of promoter induction in the recombinant plasmid. To determine which genes from *S. thermophilus* were induced, the plasmid insert of each activated R-IVET clone was amplified using primers #18 and #19, purified, and sequenced using the same primers by Genewiz Company (Takeley, UK). The nucleotide sequences were analyzed by NCBI BLASTn using the annotated genome sequence of wild-type *S. thermophilus* LMD-9 [34]. A promoter prediction was performed on sequences obtained by using the online promoter prediction tools Softberry BPROM [39] and phiSITE Promoter Hunter [40]. 

### 2.10. Statistical Analyses

Data were analyzed using a two-way repeated-measures analysis of variance (ANOVA) followed by a Bonferroni test. The statistical analyses were performed using GraphPad Prism software 7.01 (GraphPad Software, Inc., San Diego, CA, USA). Results were expressed as means ± SEM. Differences were considered statistically significant when *p* < 0.05. Survival kinetics of the R-IVET library in the TIM-1 system were compared to that of a theoretical transit marker provided by the computer. This marker evolves according to the volume of each compartment, the rate of dilution by digestive secretions, and the chyme flow between two successive compartments, and it corresponds to a 100% survival rate. The comparison was independently performed in each compartment and at each time of sampling. For fecal batch cultures, the number of cultivable cells was determined, and a comparison was independently performed between volunteer 1 and 2 at each time of sampling.

## 3. Results

### 3.1. Construction and Validation of the R-IVET Positive Screening Tool

**Figure 3:**
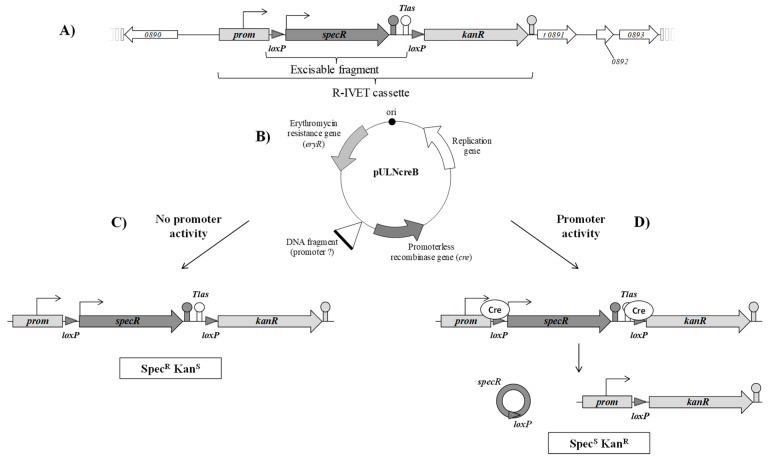
Schematic representation of positive-selection R-IVET screen in *S. thermophilus* STUL5003. The R-IVET technology consists of two elements. The first one is the chromosomal cassette (**A**) with the spectinomycin resistance gene (*specR*) flanked by two *loxP* sites followed by the promoterless kanamycin resistance gene (*kanR*). The second element is the pULNcreB plasmid (**B**, Table 1) which possesses the promoterless recombinase gene (*cre*). Cre recombinase recognizes the *loxP* sites of the cassette. (**C**) When the DNA fragment cloned upstream of the *cre* gene is without promoter activity, only the *specR* gene is expressed and the two terminators, i.e., *specR* gene and *Tlas* downstream of the *specR* gene, prevent expression of the *kanR* gene. The corresponding clone is spectinomycin-resistant and kanamycin-sensitive (Spec^R^Kan^S^). (**D**) When the DNA fragment possesses promoter activity, Cre recombinase is produced and leads to excision of the *specR* gene; the *kanR* gene can, therefore, be expressed from its own promoter. Hence, the clone is spectinomycin-sensitive and kanamycin-resistant (Spec^S^Kan^R^).

The R-IVET positive screen was designed on the basis of the strategy developed for *Enterococcus faecalis* [40]. The chromosomal cassette consisting of two antibiotic resistance genes was constructed as detailed in Figure 1 and in Section 2.3 and then introduced into the locus STER_0891, encoding a putative glucose uptake permease, of the *S. thermophilus* LMD-9 genome to obtain the mutant strain STUL5003. This cassette consisted of the promoter (*prom*) of the kanamycin resistance gene *kanR*, followed by a *loxP* site, the spectinomycin resistance gene *specR* with its own promoter, the *Tlas* terminator, another *loxP* site, and the promoterless *kanR* gene. Thus, the strain STUL5003 was resistant to spectinomycin and sensitive to kanamycin (phenotype Spec^R^Kan^S^). It was expected that, under the expression of the Cre recombinase, the *specR* gene would be excised and, consequently, the *kanR* would be correctly positioned to be expressed under the promoter *prom* (Figure 3).

The STUL5003 chromosomal cassette was sequenced to ensure that no mutation occurred during the construction. When compared with the expected sequence of the R-IVET cassette, two mutations were detected: replacement of a cytosine by a guanine in the *prom* region and deletion of a cytosine in the transcription terminator of the *specR* gene. Regarding the position of these mutations, as well as the phenotype of STUL5003 and STUL5003-*plac* strain colonies, as presented below, we concluded that these mutations had no significant impact on the functionality of our R-IVET tool.

Functionality of the R-IVET positive screen was validated using the plasmid pULNcreB-*plac* containing the *plac* promoter of lactose operon (Table 1). As this promoter is inducible by lactose and less active in the presence of glucose [23], it was expected that, following the expression of Cre recombinase, all STUL5003-*plac* strain colonies grown in the presence of lactose would lose the *specR* gene and express the *kanR* gene. Hence, this plasmid was introduced into STUL5003 strain to obtain STUL5003-*plac* strain. Sixty randomly chosen colonies from both strains were grown on five different agar media to check their antibiotic resistance phenotype: LM17 medium without antibiotic (control) or supplemented with either spectinomycin or erythromycin (to confirm the presence of pULNcreB-*plac* plasmid, which has the *ermE* gene conferring erythromycin resistance), with kanamycin, or with a mix of erythromycin and kanamycin. We observed that all 60 STUL5003-*plac* strain colonies exhibited a Spec^S^Kan^R^Ery^R^ phenotype, because they grew without antibiotic, as well as with erythromycin, kanamycin, or both, but not with spectinomycin. As expected, none of the 60 STUL5003 strain colonies was able to grow with erythromycin and/or kanamycin, while they grew with spectinomycin, as well as on control medium (Spec^R^Kan^S^Ery^S^ phenotype). The presence/absence of a *loxP–specR* excisable fragment was checked by PCR in randomly chosen colonies of STUL5003 and STUL5003-*plac* strains. In each colony (6/6) of STUL5003 strain, an amplicon of 2091 bp was observed as expected since *loxP–specR* was present, while an amplicon of 628 bp was detected in each colony (3/3) of STUL5003-*plac* strain, since *loxP–specR* was absent. Lastly, sequencing of three STUL5003-*plac* amplicons confirmed that the chromosomal cassette was deleted.

In addition to validation of R-IVET positive screening, these results also attested to a certain stability of the chromosomal cassette, since none of the 60 STUL5003 colonies lost the chromosomal cassette through a recombination between the *loxP* sites which, in fact, constituted direct repeats. Lastly, it must be underlined that, at this stage, all of the Spec^S^Kan^R^Ery^R^ R-IVET clones (almost 2500) analyzed throughout this work displayed a recombinant pULNcreB.

### 3.2. Construction of the R-IVET Genomic Library and Counterselection of Activated R-IVET Clones

The R-IVET genomic library was constructed using *Escherichia coli* TOP10 as an intermediate cloning host. Genomic DNA of *S. thermophilus* LMD-9 partially digested with *Alu*I was ligated with *Sma*I-digested pULCreB plasmid (Table 1). Then, recombinant plasmids were introduced into *E. coli* TOP10, and 72,000 clones were selected. Plasmids extracted from *E. coli* TOP10 were introduced into *S. thermophilus* STUL5003, resulting in approximately 114,600 colonies selected on LM17-ery medium. Hence, PCRs were performed on plasmids from 56 randomly chosen R-IVET recombinant clones to (i) assess the proportion of recombinant clones, and (ii) establish insert sequences to evaluate LMD-9 genome recovery. The results showed that 37.5% (21/56) of R-IVET clones possessed an insert, with an average size of 500 bp. Analyses of insert sequences revealed that 13 corresponded to simple inserts and eight corresponded to multi-inserts. In the rest of this work, about 50% of R-IVET activated clones exhibited a multi-insert. They were systematically excluded from analyzes. This probably resulted from the method used to set-up the R-IVET genomic library, since it was decided to dephosphorylate the *Sma*I-digested pULNcreB vector to avoid its self-ligation instead of dephosphorylating the inserts. According to the proportion of recombinant clones (37.5%) and of that of multi-inserts (50%), the probability that each part of *S. thermophilus* LMD-9 genome exists at least once in the form of a simple insert was estimated at 0.997, using the Clarke and Carbon formula [41]. Lastly, the corresponding fragments of simple inserts came from different parts of the *S. thermophilus* genome, and no specific genomic region appeared to be over- or under-represented.

As R-IVET technology was used to identify promoters specifically induced under simulated human digestive conditions, counterselection of R-IVET recombinant clones containing a promoter induced during their growth before introduction into one of the three gut models used in this work constituted an essential first step. Hence, preselection conditions were optimized to eliminate Spec^S^Kan^R^ clones (i.e., clones where recombinase was expressed) and select Spec^R^Kan^S^ clones (i.e., clones keeping an intact original chromosomal cassette since Cre recombinase was not expressed). R-IVET genomic library clones were grown either in milk (growth medium used for the TIM-1 system and batch cultures of human fecal microbiota) or in LM17 (Caco-2 TC7 model) and exposed to antibiotics at different times, as described in Section 2.5. The better selection condition found corresponded to exposition for at least 7 h to a mix of spectinomycin/streptomycin (*specR* conferring resistance to both antibiotics) but it seemed impossible to eliminate Spec^S^Kan^R^ clones present in initial cultures. Consequently, the Kan^R^ clones obtained at the beginning of each experiments (T0) were systematically and carefully analyzed in each of the experiments described in this work.

### 3.3. Survival Kinetics of S. thermophilus under Human Simulated Conditions

Before starting selection of activated R-IVET clones in the three complementary gut models, viability of the R-IVET genomic library under digestive conditions was established, given that *S. thermophilus* survival in the human GI tract is a key parameter to consider for further evaluation as a probiotic, according to the regulatory definition [6]. Fermented milk was chosen as a food carrier for *S. thermophilus* in TIM-1 and in fecal batch cultures, since it was previously shown to improve bacterial survival in the in vitro stomach compared to milk [18]. Furthermore, *S. thermophilus* is commonly consumed by humans via ingestion of fermented milks such as yogurt. For the Caco-2 TC7 adhesion model, a culture medium simpler than fermented milk, i.e., LM17 medium was preferred.

**Figure 4:**
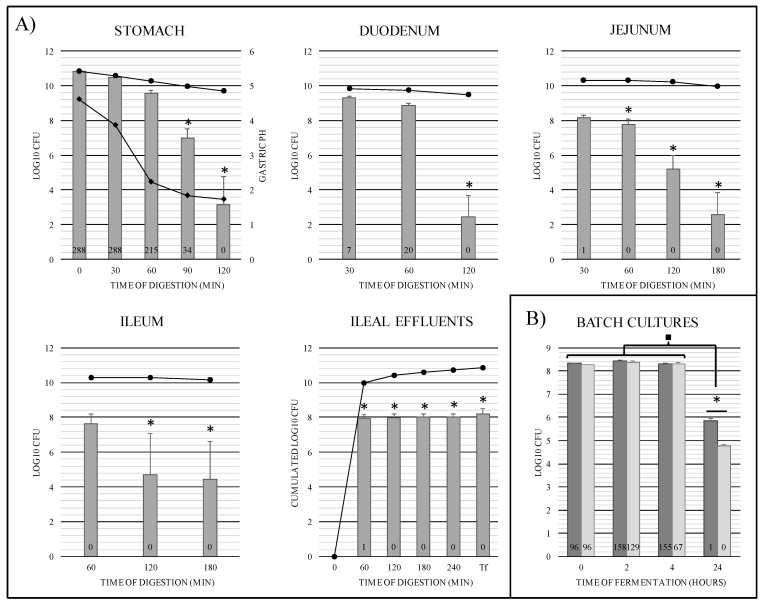
Bacterial survival and number of activated R-IVET clones obtained and analyzed in each TIM-1 compartment (**A**) and in fecal microbiota batch cultures (**B**). (**A**) In each TIM-1 compartment, data points obtained for the R-IVET library and the theoretical transit marker are represented by gray bars and black circle curves, respectively. In the stomach compartment, the black diamond curve gives the gastric pH evolution over time. Tf represents the cumulated ileal effluents plus gastrointestinal residue collected at the end of TIM-1 experiment. Values are given as means of log_10_ CFU ± SEM (*n* = 3). At each time, results for the R-IVET library were compared to those of the transit marker. Significant differences are noted by asterisks (ANOVA and Bonferroni test, *p* < 0.05). Numbers in bars correspond to the total number of activated R-IVET clones obtained and analyzed at each time point. (**B**) For batch cultures, dark-gray bars and light-gray bars represent data from volunteer 1 (man) and volunteer 2 (woman), respectively. Values are given as means of log_10_ CFU ± SEM (four technical replicates). Values at each time point were compared to those obtained at T_0_ (^■^ *p* < 0.05) and between each volunteer (* *p* < 0.05). Numbers in bars correspond to the total number of activated R-IVET clones analyzed at each time point.

In TIM-1, bacterial survival kinetics was not significantly different from that of a theoretical transit marker provided by the in vitro model during the 60 min digestion in the gastric compartment (Figure 4A). Conversely, significant bacterial mortality (*p* < 0.0001) was observed from 90 min when pH fell below 1.8, with a loss of 3 log_10_ CFU and 7 log_10_ CFU compared to the transit marker at 90 and 120 min, respectively. Monitoring of R-IVET library cell numbers in the three compartments of the small intestine showed a negative impact of digestive conditions on survival ability of these clones in post-gastric conditions (Figure 4A). The number of viable cells showed a significant decrease (*p* < 0.001) in the small intestinal compartments after 120 min of digestion, with a loss of around 8 log_10_ CFU in the duodenum and 6 log_10_ CFU in the jejunum and ileum compared to the transit marker. Bacterial cells which survived the whole digestive process were recovered in the TIM-1 ileal effluents. Final counts, i.e., when the numbers of bacterial cells in ileal effluents and gastrointestinal residue were added, reached 8.2 log_10_ CFU versus 9.2 log_10_ CFU for the wild-type strain, as previously established by Uriot et al. [18]. This could result either from interruption of the STER_0891 locus where the chromosomal cassette was introduced or from a mutation elsewhere in the genome of STUL5003 strain.

In batch cultures of human fecal microbiota, survival rates of the R-IVET library remained unchanged during the first 4 h of incubation (Figure 4B), and no significant difference was observed between the two volunteers. However, after 24 h incubation, the number of viable *S. thermophilus* R-IVET cells significantly decreased by approximately 2.5 and 3.5 log_10_ CFU for volunteers 1 and 2, respectively. The final survival rate was significantly different (*p* < 0.0001) between the two volunteers. This result suggested that the R-IVET library was not able to colonize the colon, as observed with wild-type strains in animals and humans after yogurt ingestion [17]. In previous studies in germ-free rat models, lactose was shown to significantly improve the survival capacity of *S. thermophilus* in the GI tract [21,42]. Of note, here, no supplementary nutrient or carbon source that could have been easily used by *S. thermophilus* was added to batch cultures.

Lastly, the survival ability of the R-IVET library was determined in the medium used for Caco-2 TC7 cell adhesion assays, in the same conditions as described in Figure 2, but without Caco-2 cells. Results showed no significant decrease in clone survival after 4 h incubation in DMEM medium and triton X-100 at 0.1% (Figure S1, Supplementary Materials).

### 3.4. Sequence Analyses of Initially Activated (T0) R-IVET Genomic Library Clones

**Figure 5:**
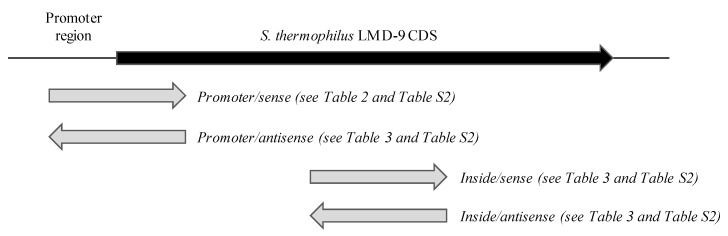
Location, orientation, and denomination of inserts of activated R-IVET clones compared to *S. thermophilus* LMD-9 genes. CDS: coding DNA sequence.

For the reasons exposed before, R-IVET clones displaying a Spec^S^Kan^R^ phenotype at T0, i.e., before their introduction in one of the three models, were selected and analyzed. From a physiological point of view, they corresponded to clones that were in a stationary growth phase, with a growth medium at about pH 4.6. Sequencing of inserts from a total of 963 clones selected revealed that about 50% consisted of multiple inserts, which were eliminated from the study. Sequencing of single inserts revealed that they originated from about 150 different genes, with certain inserts matching with at least two genes because of the presence of repeated sequences inside the inserts (Table S2, Supplementary Materials). This implies that most of the 150 genes were observed several times. Three situations were distinguished, whereby inserts included (i) a gene promoter (84 genes or 56%), (ii) a sequence located inside the promoter of a gene but on the antisense strand (eight genes or 0.05%), and (iii) a sequence located inside a gene, on either the sense or the antisense strand (Figure 5 and Table S2, Supplementary Materials). Interestingly, among the 49 promoters from T0 clones of TIM-1 and fecal batch culture, 33 were identified in both and probably correspond to genes induced during growth in milk. Lastly, 20 T0 clones were common to the three gut models, which is consistent with them corresponding to genes involved in basic cellular functions, for example, PepS aminopeptidase, subunit III of DNA polymerase III, ATP subunit of Clp protease, DNA/RNA helicase, or DNA polymerase I (Table S2, Supplementary Materials).

### 3.5. Identification of S. thermophilus Functions Induced in GI Models

After transit through TIM-1, fecal batch incubation, or adhesion to Caco-2 TC7 cells, 1461 Spec^S^Kan^R^ clones were recovered for further analysis. As for T0 samples, single or multiple inserts upstream of *cre* gene were observed. Once multiple inserts and T0 activated genes were excluded from the analysis, the resting single-insert sequence corresponded to 98 genes. Once again, some of them were found several times. For 44 genes, the insert included the promoter of a gene already annotated on the LMD-9 strain genome (Table 3), whereas the insert corresponding to the 54 other genes included an internal gene sequence (on the sense or antisense strand) or sequences located on the antisense strand of a gene promoter region (Table 4). Analysis of their sequences using promoter prediction software showed that all of them contained at least a strong signal of promoter activity, often well positioned in relation to the gene *cre,* considering that 45 bp separated the end of the insert from the beginning of gene *cre*.

Of the 44 gene promoters, 18, 20, and six were identified in TIM-1, Caco-2 TC7 cells, and fecal batch cultures, respectively (Table 3). In TIM-1, the 18 promoters were identified in the stomach compartment, mainly after 30 and 60 min digestion. In fecal batch cultures, two promoters were observed after 2 h and four promoters were observed after 4 h incubation. Interestingly, only one promoter was detected both in TIM-1 and in fecal batch cultures, while others remained gut region-specific. Functional classes of activated genes were also mainly gut model-dependent. For example, seven out of 18 genes activated in TIM-1 have a protein synthesis function and three are involved in nutrient absorption and metabolism. In Caco-2 TC7, three genes encoding cell surface proteins and two encoding competence proteins were specifically activated. Lastly, two out of six genes activated in fecal batch cultures are involved in the stress response. Of note, a few genes encoding regulators (two in TIM-1 and one in Caco-2 TC7) were also detected in R-IVET activated clones. Furthermore, four genes encoding hypothetical proteins were activated in Caco-2 TC7 compared to three in TIM-1 and only one in fecal batch cultures. Interestingly, inserts from activated R-IVET library clones located inside genes or on the antisense strand mainly belonged to genes involved in nutrient absorption and metabolism (15/55), protein synthesis (5/55), and stress response (6/55). On the remaining 23 inserts, 13 belonged to genes involved in various functions such as peptide synthesis, DNA repair, or DNA internalization (Table 4).

### 3.6. Determination of Activated Gene Variability in S. thermophilus Strains Classified According to Their Resistance to GI Stresses and Adhesion Capacity

In a previous study, we determined the capacity of 30 phylogenetically very close strains of *S. thermophilus* to resist different stresses known to prevail in the human digestive tract (including acid stress) or to adhere to HT29-MTX mucus-producing cells. This led to the identification of six distinct phenotypic classes [15]. Therefore, we investigated the variability of activated genes from the present study in strains belonging to some of these six phenotypic groups. All genes identified in activated R-IVET clones from TIM-1, fecal batch cultures, or Caco-2 TC7 cells were amplified from the genomes of selected strains (CNRZ160, CNRZ21, EBL308, EBL385, EBLST20, PB18O, PB302, PB385, PB5MJ, ST14, and ST88; see Table 1). Sequences obtained were then compared with similar ones in fully sequenced LMD-9 and LMG18311 *S. thermophilus* strains [2,34].

**Figure 6:**
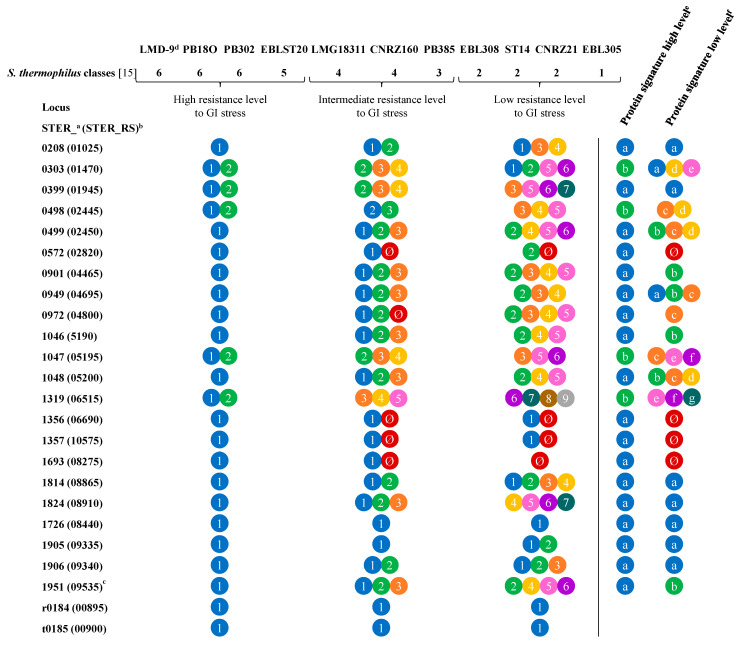
Schematic overview of allele variability of R-IVET activated genes identified in TIM-1 and fecal batch cultures among strains displaying different properties of resistance against GI stress and deduced protein signature. Colors/numbers correspond to different alleles of R-IVET activated genes that were identified. Different proteins signatures are given by different colors/letters. The full name of each gene is detailed in Table S3 (Supplementary Materials). ^Ø^ No PCR amplicon; ^a^ locus annotation from 28 January 2014; ^b^ locus annotation from 30 July 2015 from NCBI; ^c^ according to Junjua et al. [15]; ^d^ LMD-9 used as a reference strain for comparison; ^e^ types of proteins found in strains exhibiting a high level of resistance against GI stress; ^f^ types of proteins found in strains exhibiting a low level of resistance against GI stress.

As shown in Figure 6 (see full data in Table S3, Supplementary Materials), tested strains displayed three resistance levels against acid stress: high level (strains LMD-9, PB18O, PB302, EBLST20, and EBL385), low level (strains EBL308, ST14, and CNRZ21), and intermediate level (others) [15], with the most sensitive being strain CNRZ21. Out of the 24 genes tested and the 22 corresponding proteins, six (four proteins and two genes) were identical in all selected strains. On the contrary, 18 predicted proteins varied, among which 12 are encoded by genes specifically activated in TIM-1, three are encoded by genes specifically activated in fecal batch cultures, and three are encoded by genes activated in both models. They are involved in various functions such as nutrient absorption and metabolism, regulation of transcription, or stress response. Some of them correspond to hypothetical proteins (Table 3). Interestingly, strains displaying a high resistance level to acidity, especially LMD-9, PB18O, PB302, and EBLST20, shared greater similarity in allele profile than the most sensitive ones (EBL308, ST14, and CNRZ21). A protein signature could be deduced from a comparison of acid-resistant strains, which seemed less obvious for sensitive strains where more variability was observed (Figure 6 and Table S3, Supplementary Materials).

According to Junjua et al. [15], selected strains displayed also different adhesion capacities to HT29-MTX cells: high capacity for LMD-9, ST88, EBLST20, and LMG18311, intermediate capacity for PB18O and EBL385, and low capacity (unable to adhere) for CNRZ21 and PB5MJ. No clear difference was observed between strains depending on their adhesion capacity (Table S4, Supplementary Materials). Patterns obtained for the very adherent strains LMD-9, ST88, and EBLST20 were very similar, as well as similar to that of weakly adherent strains such as PB5MJ. Furthermore, the pattern obtained for the very adherent strain LMG18311 was very similar to that of the weakly adherent strain CNRZ21. This absence of variability might result from differences in cell models used in our work and Junjua et al. [15].

## 4. Discussion

The aim of this work was to better understand the physiological status of *S. thermophilus* during its passage through the human digestive environment. To reproduce the different niches found in the human GI tract, three complementary in vitro models were used, namely, the TIM-1 (stomach and small intestine), fecal batch cultures (colon), and the Caco-2 TC7 cells (interaction with intestinal epithelial cells). Among available approaches that allow the following of bacterial gene expression, we decided to use the R-IVET technology, which is the only one among IVET technologies that functions as a genetic screen [25]. The main advantage of this technology is that it makes it possible to identify genes specifically expressed in complex media, such as the human digestive environment, including transiently and locally expressed genes, at the level of the individual bacterium [25]. R-IVET also bypasses the difficult acquisition step of high-quality RNAs from complex environments required in other techniques (especially in environments with a complex microbial background as found in the human colon). First, we significantly improved the R-IVET tool applied to *S. thermophilus* LMD-9, through the construction and validation of a positive screening of activated clones. The LMD-9 reference strain was chosen for three main reasons: (i) availability of its genome sequence, (ii) ability to resist various stresses prevailing in the GI tract, and (iii) adherence to intestinal epithelial cells [15,16,18,34]. Then, we identified specifically activated genes in each digestive condition and observed that some of them varied across *S. thermophilus* strains showing different levels of resistance against GI stresses.

The chromosomal cassette we constructed allows the identification of activated genes through excision of the first reporter gene *specR*, leading to the activation of the second reporter gene *kanR*. Furthermore, by adding erythromycin to the selection medium already containing kanamycin, the activated Spec^S^Kan^R^ clones could be selected while ensuring the stability of the R-IVET plasmid. During this work, about 2500 clones displaying the Spec^S^Kan^R^Ery^R^ phenotype were analyzed. In each of them, a precise excision of the *specR* gene, through recombination between the *lox* sites flanking it, was observed, and an insert located upstream the *cre* gene was present, even though our genomic R-IVET library contained only 37.5% recombinant clones. This result provides a strong argument for the high stability of the constructed chromosomal cassette and the efficiency of designed screening approach. Lastly, the probability that genomic R-IVET library covered the entire LMD-9 genome was very high (0.997).

Identification of genes specifically activated under human simulated digestive conditions using R-IVET technology implies the elimination of any recombinant clone containing a promoter induced during its growth culture (i.e., before introduction in the tested environment). Despite our efforts, it was not possible to counterselect all Spec^S^Kan^R^ clones before introduction of the R-IVET library in each GI model, leading us to systematically analyze all these clones. This might result from the limited number of antibiotic resistance genes that can be efficiently used in *S. thermophilus* and constitutes a limitation of our R-IVET tool. Nevertheless, as shown in Table S2 (Supplementary Materials), inserts of T0 activated R-IVET clones often correspond to basic cellular functions expected to be activated during library growth, as bacteria have to display an active metabolism to multiply. Hence, promoters of the corresponding genes cloned upstream the *cre* gene are obviously activated.

**Figure 7:**
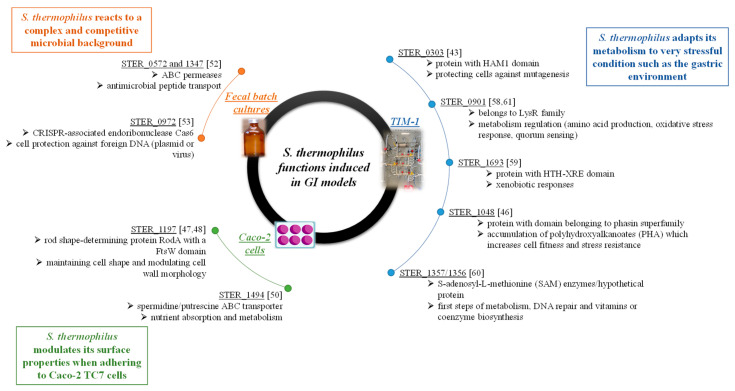
Synthetic view of main genes from *S. thermophilus* LMD-9 specifically induced in each GI models and their possible involvement in cell functions [43,46,47,48,50,52,53,58,59,60,61].

Throughout in vitro experiments, among the 2424 Spec^S^Kan^R^ clones analyzed, 128 promoters were detected, including 44 specifically activated in the GI models. All of these 44 promoters, expect one corresponding to a gene encoding a hypothetical protein of the phasin superfamily, appeared to be GI model-specific, strengthening the argument that they do not correspond to false positives. Furthermore, in each in vitro model, these promoters were represented several times among activated inserts. Thus, the first conclusion is that *S. thermophilus* LMD-9 is metabolically active and can finely adapt its metabolism to each of the tested digestive conditions. All of the 18 promoters identified in TIM-1 were isolated from the gastric compartment, and no promoter was identified in the small intestine. This could be linked to a survival loss due to acidic pH in the stomach and high bile salt concentrations in the proximal small intestine. as the number of activated R-IVET clones that can be recovered is already weak, the significant drop in genomic library survival in the gastric and duodenal compartments might have contributed to the non-selection of activated clones. In the gastric compartment, *S. thermophilus* activated a higher number of genes involved in nutrient absorption and metabolism and in protein synthesis (10/18) compared to Caco-2 TC7 cells (3/20) and the colon model (2/6). Interestingly, while the gastric compartment was expected to be very stressful because of acidic conditions, only one gene (STER_0303) involved in stress response was identified (Figure 7). This gene might be involved in protecting cells against mutagenesis in relation to the HAM1 domain found in the last 200 amino-acid residues of the protein [43]. Detection of such a low number of stress-related genes could also result from adaptation to acidic conditions of the R-IVET library during its growth in milk, since, at the end of fermentation, pH was still low (pH 4.6). Therefore, genes known to be involved in acidic stress resistance such as GroEL, GroES, or Hsp [44,45], activated from pH 5, were likely expressed during the growth phase, and the corresponding clones were eliminated during the counterselection step applied before TIM-1 inoculation. Of interest, an insert belonging to the *groES* gene was detected in an activated R-IVET clone; however, as it was a multi-insert clone, it was not included in the study. Even if there was only one stress protein induced, analysis of other genes activated during the TIM-1 gastric phase suggests an adaptation of the bacterium to very stressful conditions (Figure 7). As an example, the gene STER_1048 encodes a protein that displays a domain belonging to the superfamily of phasins, which are involved in many bacteria during formation and intracellular accumulation of polyhydroxyalkanoates under adverse conditions, which allows cells increasing their fitness and resistance to stresses [46].

Genes induced during adhesion assays suggest that *S. thermophilus* modulated its surface properties to adhere to Caco-2 TC7 cells (Figure 7). Three genes encoding cell surface proteins were induced during cell adhesion, two of them having an unknown function. The third was a cell membrane division protein designated as rod shape-determining protein RodA (STER_1197). This protein contains the FtsW domain [47], which appears to be required in crossing peptidoglycan precursors outside the cell membrane and for peptidoglycan biosynthesis, thus being involved in maintaining cell shape and modulating cell-wall morphology [48]. Furthermore, it has been shown that the RodA protein is essential for the cell viability of *Bacillus subtilis* and to maintain cell surface shape [49]. Three genes involved in nutrient absorption and metabolism were also induced; among them, one (STER_1494) encodes an ABC transporter [50]. Even if its role has to be further documented, it must be noted that a significant decrease in adhesion of *B. bifidum* to Caco-2 TC7 cells has been observed after deletion of ABC transporters encoding genes located at the outer cell surface [51].

Only six promoters were identified in batch cultures of human fecal microbiota. Two of them correspond to genes involved in protein synthesis, two encode stress proteins (STER_0572 and STER_1347 genes encoding two ABC permeases possibly involved in antimicrobial peptide transport; see [52]), and two encode proteins of the CRISPR immune system, one being an hypothetical protein while the other is the CRISPR-associated endoribonuclease Cas6. The activation of stress proteins and proteins from the CRISPR system involved in cell protection against foreign DNA, such as plasmid or virus DNA [53], suggests that *S. thermophilus* reacts to an environment where competition with other gut microorganisms is very hard (Figure 7).

Moreover, 54 unique inserts allowing the expression of the *cre* gene but not corresponding to an already annotated promoter region were revealed in gut models. There were three different cases for the location of insert sequence allowing Cre recombinase expression: (i) inside a gene and in the sense orientation, (ii) inside a gene but in the antisense orientation, and (iii) in the promoter region of a gene but on the antisense strand. All these inserts ere carefully analyzed using several predictive promoter software. In any case, there was at least one part of the sequence, located near the insert extremity and, hence, correctly positioned, that could serve as a promoter. Furthermore, analysis of the sequence located downstream revealed, in certain cases, the presence of small ORFs. The *S. thermophilus* genome is known to contain small ORFs encoding peptides involved in the regulation of response such as quorum sensing [54,55]. Thus, if their promoter activity is confirmed, these inserts could belong to regions containing small ORFs, not yet considered during genome annotation. Furthermore, sequences located inside a gene but in the antisense orientation could correspond to promoters of potential noncoding antisense RNA, as previously suggested in other studies using R-IVET technology, e.g., in *L. plantarum* [25] or *Enterococcus faecalis* [56,57]. As mentioned before, no Spec^S^Kan^R^ clone obtained throughout this work was devoid of an insert. However, we cannot formally exclude that at least certain inserts corresponded to false positives as proposed by Koguchi et al. [27]. If we rule out the false positive hypothesis, then these data suggest the R-IVET technology as helpful for a better understanding of gene regulation, with potential use in genome annotation of a targeted strain such as *S. thermophilus* LMD-9.

Lastly, we established the allelic form of the 44 genes revealed in GI models and deduced corresponding proteins in a collection of phylogenetically very close *S. thermophilus* strains [15]. These strains were previously described as displaying different survival capacities to known GI stresses (such as acidic pH, bile salts, and oxidation stress) and varied adhesion capacity, although, in this last case, adhesion was determined using HT-29 MTX and not Caco-2 TC7 cells. For the 24 genes activated in TIM-1 and fecal batch cultures, comparison of the corresponding proteins between strains having a high level and a low level of resistance to GI stresses allowed us to draw a protein signature for the two categories. The next step will be to determine their role in *S. thermophilus* resistance to GI conditions by making mutants and performing further experiments in TIM-1 and batch culture models. The ultimate goal of this study is to apply R-IVET technology to identify selection markers of GI resistance that will be helpful in the screening of *S. thermophilus* strains with potential probiotic properties.

## Figures and Tables

**Table 1:** Bacterial strains and plasmids used in the present study.

Strain or Plasmid	Relevant Markers and Characteristics	Reference or Source
Strains		
*S. thermophilus* CNRZ160	Wild-type strain whose genome has been entirely sequenced	STH_CIRM_16 CNRZ collection
*S. thermophilus* CNRZ21	Wild-type strain	CNRZ collection, Nancy subclone
*S. thermophilus* EBL308	Wild-type strain	Laboratory collection
*S. thermophilus* EBL385	Wild-type strain	Laboratory collection
*S. thermophilus* EBLST20	Wild-type strain	Laboratory collection
*S. thermophilus* LMD-9	Wild-type strain whose genome has been entirely sequenced	ATCC BAA-491 [34]
*S. thermophilus* LMG18311	Wild-type strain whose genome has been entirely sequenced	ATCC BAA-250 [2]
*S. thermophilus* PB18O	Wild-type strain	Laboratory collection
*S. thermophilus* PB302	Wild-type strain	Laboratory collection
*S. thermophilus* PB385	Wild-type strain	Laboratory collection
*S. thermophilus* PB5MJ	Wild-type strain	Laboratory collection
*S. thermophilus* ST14	Wild-type strain	Laboratory collection
*S. thermophilus* ST88	Wild-type strain	Laboratory collection
*S. thermophilus* STUL5002	LMD-9 derivative with the *prom-loxP-kanR* fragment in the STER_0891 locus (STER_RS04415)	Present study
*S. thermophilus* STUL5003	LMD-9 derivative with the *prom-loxP-specR-loxP-kanR* fragment in the STER_0891 locus (STER_RS04415)	Present study
*S. thermophilus* TIL1193	LMD-9 *feoB::aphA3*	[33]
*E. coli* TOP10	One Shot^®^ TOP10 chemically competent *E. coli* cells	Invitrogen
Plasmids		
pULNcreB	pG^+^host9^TR^ derivative containing promoterless recombinase *cre* gene	[23]
pSET4S	Replication function of pG^+^host3 and pUC19	[35]
pULNcreB-*plac*	pULNcreB derivative containing the promoter of the lactose operon *plac* cloned in the *Bgl*II site upstream of *cre*	[23]

*S. thermophilus* strains came either from CNRZ (Centre National de Recherches Zootechniques, INRA, Jouy-en-Josas, France) or ATCC (American Type Culture Collection, Manassas, VA, USA) collections or were isolated in our laboratory from either yogurt or cheese.

**Table 2:** Parameters of TIM-1 gastrointestinal model used to simulate milk digestion by a healthy human adult (adapted from [18]).

Compartment	Volume (mL) at Initial Time	pH/Time (min)	Digestive Secretions	t_1/2_ (min)	β Coefficient
Stomach	310	2/0, 4.2/20, 2.8/40, 2.1/60, 1.8/90, 1.7/120, 1.7/240	0.25 mL/min of pepsin (500 U/mL) 0.25 mL/min lipase (75 U/mL) or HCl (3M) if necessary	30	1
Duodenum	40	Maintained at 6.0	0.5 mL/min of bile porcine extract (4% *w*/*w* during the first 30 min of digestion and then 2% *w*/*w*) 0.25 mL/min of porcine pancreatin (14.1% *w*/*w*) 0.25 mL/min of intestinal electrolyte solution or NaHCO_3_ (1 M) if necessary		
Jejunum	105	Maintained at 6.8	0.25 mL/min of NaHCO_3_ (1 M) if necessary		
Ileum	110	Maintained at 7.2	0.25 mL/min of NaHCO_3_ (1 M) if necessary	160	1.6

A power exponential equation (f = 1 − 2^−(t/t1/2)β^ where f represents the fraction of meal delivered, t is the time of delivery, t_1/2_ is the half-time of delivery, and β is a coefficient describing the shape of the curve) was used for the computer control of gastric and ileal deliveries.

**Table 3:** Inserts from activated R-IVET library clones identified under simulated human digestive conditions and found in the annotated *S. thermophilus* LMD-9 genome.

Class	Locus STER_ ^a^ (STER_RS) ^b^	Gene Description	Position of the Insert on LMD-9 Genome (nt)	TIM-1	Caco-2 TC7	Fecal Batch Cultures
Cell surface proteins	0314 (01525)	Membrane protein (predicted)	270,262–270,470			
	0758 (03720)	Membrane protein (predicted)	690,246–690,721			
	1197/1196 * (05905/05900)	Rod shape-determining protein RodA/IS5 family transposase	1,106,940–1,104,772			
Competence proteins	0406 (01985)	Competence protein	355,852–357,052			
	1477 (07260)	Type II CRISPR RNA-guided endonuclease Cas9	1,384,541–1,380,985			
Nutrient absorption and metabolism	0399 (01945)	Branched-chain amino-acid ABC-type transport system, permease component	350,432–350,646	S30		
	0498/0499 (02445/02450) *	Predicted amidohydrolase/transaminase	445,501–447,041	S30 S90		
	0949 (04695)	Zn-dependant alcohol dehydrogenase	879,316–879,396	S30		
	1371 (06720)	Galactokinase	1,281,820–1,280,237			
	1494 (07350)	ABC transporter permease	1,399,529–1,399,241			
	1743 (08515)	Branched-chain amino-acid transporter	1,633,154–1,633,062			
Protein biosynthesis	0208 (01025)	30S ribosomal protein S15	176,108–176,450	S30 S60		
	r0082/t0083 (00415/00420)	rRNA-5S ribosomal RNA/tRNA-Val	71,512–71,726	S30		
	r0071/t0072 (00830/00835)		145,721–145,935			
	r0022/t0023 (00110/00115)		23,850–24,064			
	r0412/t0413 (02015/02020)		364,159–364,373			
	r0184/t0185 (00895/00900)	rRNA-5S ribosomal RNA/tRNA-Asn	151,527–151,756	S30		
	r1781/t1780 (08710/08705)		1,662,586–1,662,357			
	1726 (08440)	30S ribosomal protein S18	1,616,742–1,616,519			M4
	1906/1905 * (09340/09335)	50S ribosomal protein L4	1,768,760–1,767,556			M4
Regulators	0583/0582 * (02870/02865)	DNA-binding response regulator/Predicted signal transduction protein with C-terminal HATPase domain	531,843–530,986			
	0901 (04465)	LysR family transcriptional regulator	833,783–832,546	S30		
	1693 (08275)	Trancriptional regulator (helix–turn–helix XRE-family like protein)	1,582,297–1,583,056	S30 S60		
Stress response	B1 (09830)	Acid-shock protein (Hsp20)	1282–301			
	0303 (01470)	Noncanonical purine NTP pyrophosphatase	261,471–261,718	S30		
	0572 (02820)	ABC permease transporter	517,756–517,057			W2
	1347 (06650)	ABC permease transporter	1,253,799–1,254,182			W2
Hypothetical proteins	0004 (00020)	Hypothetical protein	3416–4150			
	0711/0712 (03500/03505)	CRISPR-associated protein, Cas 2 family/hypothetical protein	647,294–648,230			
	0807 (03965)	Hypothetical protein	741,250–739,908			
	1048/1047/1046 * (05200/05195/05190)	Hypothetical protein (phasin protein superfamily)/predicted unusual protein kinase/hypothetical protein	974,176–971,354	S30		W4
	1319 (06515)	Hypothetical protein (nitroreductase-like protein family)	1,228,286–1,227,463	S30 S60		
	1968 (09625)	Hypothetical protein	1,818,680–1,819,008			
Other functions and pseudogenes	0574 (02830)	Pseudo, partial start: fibronectin-binding protein	518,245–518,397			
	0811 (03990)	Transposase	746,827–746,519			
	0972 (04800)	CRISPR-associated endoribonuclease Cas6	897,070–898,203			M4
	1074 (05335)	Pseudogene: voltage-gated chloride channel	998,385–997,070			
	1357/1356 * (10575/06690)	Hypothetical protein/KxxxW cyclic peptide radical SAM maturase	1,261,748–1,260,668	S60		
	1485 (07300)	Arsenate reductase family protein	1,391,653–1,391,193			
	1814 (08865)	2,3,4,5-Tetrahydropyridine-2,6-dicarboxylate *N*-acetyltransferase	1,695,365–1,694,980	S60		
	1824 (08910)	tRNA-binding protein	1,701,896–1,701,414	S60		
	1859 (09090)	Pseudogene: alcohol dehydrogenase	1,740,375–1,739,341			
	1952 (09545)	Pseudogene: 5-methyltetrahydrofolate-homocysteine methyltransferase	1,810,490–1,810,368			

A cell in blue, orange or green means that a promoter has been activated specifically in the TIM-1, Caco-2 TC7 or Fecal batch cultures, respectively. ^a^ Locus annotation from 28 January 2014; ^b^ locus annotation from 30 July 2015 from NCBI; * the insert is correctly positioned to detect the expression of promoter regions from two adjacent genes, with the possibility of an operon. S30, S60, and S90 denote genes identified in the TIM-1 stomach compartment at 30 min, 60 min, and 90 min, respectively. M4 denotes genes identified in fecal batch cultures from the male volunteer at 4 h. W2 and W4 denote genes identified in fecal batch cultures from the female volunteer at 2 h and 4 h, respectively.

**Table 4:** Inserts from activated R-IVET library clones identified under simulated human digestive conditions and not associated with annotated promoter regions from *S. thermophilus* LMD-9 genome.

Class	Locus STER_ ^a^ (STER_RS0) ^b^	Gene Description	Location and Direction Compared to the Gene ^c^	Position of the Insert on LMD-9 Genome (nt)	TIM-1	Caco-2 TC7	Fecal Batch Cultures
Nutrient absorption and metabolism	0157 (0760)	PFL family protein	Inside/sense	129,819–130,812	S60		
	0391 (1905)	Cysteine desulfhydrase	Inside/sense	345,914–346,179			
	0429 (2100)	3-Oxoacyl-ACP synthase III	Promoter/antisense	374,511–374,069			
	0445 (2180)	1-Phosphofructokinase	Promoter/antisense	389,930–389,309			W4
	0537 (2635)	*N*-Acetylglucosamine-6-phosphate deacetylase	Inside/antisense	483,279–483,109	S60		
	0654 (3225)	Ferrous iron transport protein B	Promoter/antisense	594,581–593,569			M4
	0713 (3510)	Phosphate ABC transporter substrate-binding protein	Inside/sense	651,008–650,690			
	1009 (4970)	ABC-type phosphate transport system, permease component	Inside/sense	929,980–930,137			
	1293 (6375)	Multidrug ABC transporter ATP-binding protein	Promoter/antisense	1,201,333–1,202,350			
	1408 (6925)	Peptide ABC transporter permease	Inside/sense	1,316,650–1,316,193	S60		
	1478 (7265)	Phosphoserine phosphatase SerB	Inside/sense	1,384,541–1,384,484			
	1487 (7310)	3-Phosphoglycerate dehydrogenase	Inside/sense	1,392,951–1,392,865	S30		
	1558 (7660)	ATPase	Inside/sense	1,462,899–1,462,760			
	1566 (7700)	Dihydroxyacid dehydratase	Inside/antisense	1,468,250–1,469,229			
	1793 (8770)	Glutamate–tRNA ligase	Inside/sense	1,677,638–1,677,046 1,677,399–1,677,046	S30		
Protein biosynthesis	0131 (0650)	Zinc ribbon domain-containing protein	Inside/sense	107,081–107,488			W2
	0313 (1520)	tRNA (uridine(34)/cytosine(34)/5-carboxymethyl aminomethyluridine(34)-2’-*O*)-methyltransferase TrmL	Inside/sense	270,267–270,471	S60		
	0368 (1785)	Serine–tRNA ligase	Inside/sense	322,147–321,141			
	0423 (2070)	23S rRNA (uracil-5-)-methyltransferase RumA	Inside/sense	368,157–369,181	S30 S60		M2
	0783 (3840)	Isoleucine–tRNA ligase	Inside/sense	715,785–716,798			
Regulators	0216 (1050)	Adaptor protein MecA	Promoter/antisense	182,455–181,444			
	1965 (9615)	Pseudogene: PadR family transcriptional regulator	Promoter/antisense	1,818,077–1,817,746	S60		
Stress response	0471 (2315)	ABC transporter	Inside/sense	418,458–419,267			M4
	0520 (2550)	F0F1 ATP synthase subunit gamma	Inside/sense	467,311–467,205	S60		
	1293 (6375)	Multidrug ABC transporter ATP-binding protein	Promoter/antisense	1,201,333–1,202,350			
	1348 (6655)	Peptide ABC transporter ATPase	Inside/antisense	1,255,962–1,255,041			
	1444 (7105)	NAD(P)-dependant oxidoreductase	Inside/antisense	1,352,291–1,352,194	S60		
	1470 (7230)	Peroxiredoxin	Inside/sense	1,375,130–1,374,976			W2
Hypothetical protein	0290 (-)	Annotation change: no similar match	Promoter/antisense	253,078–252,966			
	1642 (8055)	Hypothetical protein	Inside/sense	1,534,778–1,534,351	S30 S90		
	1724 (8430)	Membrane protein	Inside/sense	1,614,761–1,614,899			W2
Other functions	0055 (0280)	Transposase	Inside/antisense	44,609–43,805			
	0102 (0510)	Transposase	Promoter/antisense	80,702–80,891			
	0237 (1165)	Pseudogene	Inside/antisense	202,301–201,856			
	0335 (1630)	Peptide synthetase	Inside/sense	287,711–288,710			M2
	0336 (1630)	NUDIX family hydrolase	Promoter/antisense	289,981–289,188	S30		
	0352 (1710)	Ribosomal RNA small subunit methyltransferase G	Inside/sense	308,825–308,525			
	0378 (1835)	Transporter	Inside/antisense	331,510–331,327			
	0569 (2805)	ISL3 family transposase	Promoter/antisense	513,865–513,496	S30 S60		
	0631 (3095)		Promoter/antisense	573,221–573,608	S30 S60		
	0849 (4180)		Promoter/antisense	783,047–783,434	S30 S60		
	1162 (5735)		Promoter/antisense	1,071,215–1,071,610	S30 S60		
	1556 (7650)		Promoter/antisense	1,460,020–1,460,416	S30 S60		
	0571 (2815)	Pseudogene	Inside/antisense	515,583–516,195			
	0853 (4200)	Glycosyl transferase family 3	Promoter/antisense	785,950–785,039	S60		
	0927 (4590)	FADH(2)-oxidizing methylenetetrahydrofolate--tRNA-(uracil(54)-C(5))-methyltransferase TrmFO	Inside/sense	858,330–858,443			
	1122 (5550)	DNA-directed RNA polymerase subunit beta	Inside/antisense	1,036,085–1,036,594			
	1179 (5820)	DNA repair protein RecN	Inside/sense	1,089,736–1,089,641			
	1355 (6685)	Transporter	Inside/sense	1,259,390–1,259,212			
	1356 (6690)	KxxxW cyclic peptide radical SAM maturase	Inside/sense	1,260,895–1,260,614			W2
	1520 (7480)	DNA internalization-related competence protein ComEC/Rec2	Inside/antisense	1,425,795–1,426,019	S30		
	1760 (8600)	Transposase	Inside/antisense	1,645,573–1,645,345			
	1921 (9415)	ABC transporter ATP-binding protein	Promoter/antisense	1,784,222–1,783,251	S30 S60		
	1978 (9670)	tRNA uridine-5-carboxymethylaminomethyl (34) synthesis enzyme MnmG	Inside/antisense	1,828,970–1,829,158			W4

A cell in blue, orange or green means that a promoter has been activated specifically in the TIM-1, Caco-2 TC7 or Fecal batch cultures, respectively. ^a^ Locus annotation from 28 January 2014; ^b^ locus annotation from 30 July 2015 from NCBI; ^c^ see Figure 5 for term explanations. S30, S60, and S90 denote genes identified in the TIM-1 stomach compartment at 30 min, 60 min, and 90 min, respectively. M2 and M4 denote genes identified in fecal batch cultures from male volunteer at 2 h and 4 h, respectively. W2 and W4 denote genes identified in fecal batch cultures from female volunteer at 2 h and 4 h, respectively.

## Data Availability

Not applicable.

## Supplementary Materials

The following are available online at https://www.mdpi.com/article/10.3390/microorganisms9061113/s1: Figure S1. Survival of the R-IVET library in adhesion experiment conditions; Table S1. Oligonucleotides used for the constructions, verification of chromosomal R-IVET cassette, sequencing of inserts from activated clones, and amplification of activated genes in various *S. thermophilus* strains; Table S2. Inserts from activated R-IVET clones identified at T0 in the different gut models; Table S3. Allele variability of R-IVET activated genes identified in TIM-1 and fecal batch cultures; Table S4. Allele variability of R-IVET activated genes identified in Caco-2 TC7 cells.
