# Supplementary material for: Identification of Streptococcus thermophilus Genes Specifically Expressed under Simulated Human Digestive Conditions Using R-IVET Technology
Source: Microorganisms. 2021 May 21;9(6):1113. doi: 10.3390/microorganisms9061113 (PMC8224003; doi:10.3390/microorganisms9061113)
Supplement: Supplementary file 1 [file microorganisms-09-01113-s001.zip › microorganisms-1232892-supplementary.pdf]

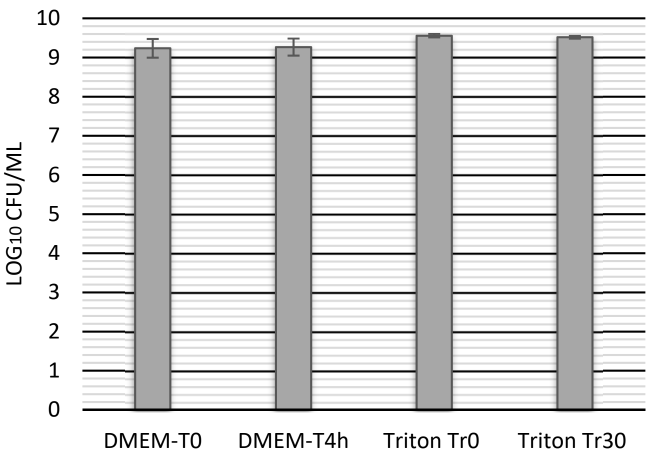

Figure S1. Survival of the R-IVET library in adhesion experiment conditions. Data represent means ± SEM from two independent experiments (n = 2) expressed by $\log_{10}$ CFU/mL viable bacterial population level determined by plate count.

Table S1. Oligonucleotides used for the constructions, verification of chromosomal R-IVET cassette, sequencing of inserts from activated clones and amplification of activated genes in various *S. thermophilus* strains

#	Sequence 5'→3'	Characteristics
**1**	ATCCGTAAAACCATCAAATCTTAATT	Amplification of the UP#1 fragment from *S. thermophilus* LMD-9 with #2 (1204 pb) [56.9°C]<br>Used in the first overlapping PCR with #8 (3783 pb) [†]
**2**	TCCCCAAGCAAAAATTGGAAC	Used with #1
**3**	TCCAATTTTTGCTTGGGGACCAGCGAACCATTTGAG	Amplification of the promoter (prom) of kanamycin resistance gene from *S. thermophilus* TIL1193 with #4 (525 pb) [56.9°C]
**4**	TCTACAGTATTTAAAGATACCCC	Used with #3
**5**	GTATCTTTAAATACTGTAGA***ATAACTTCGTATAGCATACATTATACGAAGTTAT***AAACAGGAA*	Amplification of the promoterless kanamycin resistance gene (*kanR*) from *S. thermophilus* TIL1193 with #6 (1032 pb) [56.9°C]
**6**	GTTGCGGATGTACTTCAGAAAAG	Used with #5, #11 and #17
**7**	TTTCTGAAGTACATCCGCAACGTAATGTTTGGTGTAGGTAATA	Amplification of the DOWN#1 fragment from *S. thermophilus* LMD-9 with #8 (1146 pb) [62.7°C]
**8**	TTAAACCAATCTGTCCTCGGG	Used with #1 and with #7
**9**	GGAACAAAAGCACCCACACT	Confirmation of *prom-loxP-kanR* presence in *S. thermophilus* STUL5002 and sequencing with #10 (4319 pb) [58°C]
**10**	TTAGGCTGGATGGCATAACC	Used with #9
**11**	CTACGAGACCTACTTAGCGC	Amplification of the UP#2 fragment from *S. thermophilus* STUL5002 with #12 (1088 pb) [62.7°C]<br>Used in the second overlapping PCR with #6 (3421 pb) [†]
**12**	CTAGTTATCTACACGACGGGGAGTCAATAACTTCGTATAATGTATGC	Used with #11
**13**	TGACTCCCCGTCGTGTAGATAAC	Amplification of the spectinomycin resistance gene (*specR*) from pSET4S and sequencing of the *S. thermophilus* STUL5003 cassette with #14 (1200 pb) [62.7°C]
**14**	CGCTACGATAACGCCTGTTT	Used with #13
**15**	AAACAGGCGTTATCGTAGCGGCTTCTGGAGCTCAATTGAA	Amplification of the *las* operon transcriptional terminator *Tlas* with #16 (252 pb) [62.7°C]
**16**	CTAAAGCTGACGGGGTAAAC	Used with #15
**17**	GTTTACCCCGTCAGCTTTAG***ATAACTTCGTATAGCATACATTATACGAAGTTAT***AAAGAGGAA*	Amplification of the *loxP*-DOWN#2 fragment from *S. thermophilus* STUL5002 with #6 (947 pb) [62.7°C]
**18**	GCTTCTGGAGCTCAATTGAAAG	Amplification of the insert in the recombinant plasmid derived from pULNcreB with #19 (variable but >460 pb) [58°C]
**19**	ATTCAACTTGCACCATGCCG	Used with #18
**20**	CGAAATAAGAGTAATATAGAG	Sequencing of the *S. thermophilus* STUL5003 cassette with #21 (490 pb) [58°C]
**21**	TAGTATCGACGGAGCCGATT	Used with #20
**22**	CCAGGACAATAACCTTATAGC	Sequencing of the *S. thermophilus* STUL5003 cassette with #23 (536 pb) [58°C]<br>Test for presence or absence of the *loxP-specR* fragment in the R-IVET cassette with #29 (with *loxP-specR*: 2091 pb; without *loxP-specR*: 628 pb) [58°C]
**23**	TCCTCCTCACTATTTTGATTAGTACC	Used with #22

**24**	AATAGGTACTAATCAAAATAGTGAGG	Sequencing of the *S. thermophilus* STUL5003 cassette with #25 (433 pb) [58°C]
**25**	TTTGCTTGGTAAAGCATTATGG	Used with #24
**26**	GGAGAGAATATTGAATGGACTAATG	Sequencing of the *S. thermophilus* STUL5003 cassette with #27 (455 pb) [58°C]
**27**	TCTAAAGCTGACGGGGTAAA	Used with #26
**28**	TATAGTTTACCCCGTCAGCTTTAG	Sequencing of the *S. thermophilus* STUL5003 cassette with #29 (445 pb) [58°C]
**29**	GAAAGAGCCTGATGCACTCC	Used with #22 and #28
**30**	CTCATGAGTGAGGCCGATG	Sequencing of the *S. thermophilus* STUL5003 cassette with #31 (484 pb) [58°C]
**31**	CAGGCTTGATCCCCAGTAAG	Used with #30
**32**	TGGTATGACATTGCCTTCTG	Sequencing of the *S. thermophilus* STUL5003 cassette with #33 (426 pb) [58°C]
**33**	TGCAAGTAAAATTGCACCTGTT	Used with #32
**34**	TTTCAAAAACAAGTGAAATCTTGA	Sequencing of the STER_0004 locus with #35 (433 pb) [58°C]
**35**	AAGTGACGTCCGTAATAGCTG	Used with #34
**36**	GCTCGAGGACTTATCCAACG	Sequencing of the STER_r0184/t0185 loci with #37 (298 pb) [58°C]
**37**	TACTCCGCCAGTAGGACTCG	Used with #36
**38**	CAAGTTTAGTTGGATTTTCCTAGC	Sequencing of the STER_0208 locus with #39 (613 pb) [58°C]
**39**	GCTGTTTTGAACTTGGGCTAA	Used with #38
**40**	CCACAGCTAGTGTGCTTGGT	Sequencing of the STER_0303 locus with #41 (637 pb) [58°C]
**41**	TAGTTTCCGCCTTCAAGCGT	Used with #40
**42**	CGAAGGAAAAACCAAGGAGTT	Sequencing of the STER_0303 locus with #43 (713 pb) [58°C]
**43**	TGGATACCTTCCCAAACAGAA	Used with #42
**44**	GAAATCCATAAATTAAGAACCTTGTCA	Sequencing of the STER_0314 locus with #45 (974 pb) [58°C]
**45**	CGTGTCAGTCCTGCTGGTAA	Used with #44
**46**	AAAACTTTGTTGGGGTTACTGG	Sequencing of the STER_0399 locus with #47 (873 pb) [58°C]
**47**	AAGAACAGCGGCAACAAAAG	Used with #46
**48**	CACGTTCATTCCCACAATCA	Sequencing of the STER_0399 locus with #49 (712 pb) [58°C]
**49**	TGTCCGGAGAAACCAAGAAC	Used with #48
**50**	GCAAGAGTGATTGGCCTTGT	Sequencing of the STER_0406 locus with #51 (1103 pb) [58°C]
**51**	CGCTGCTTCTTAATCTGGTG	Used with #50
**52**	CGTTTCTTTTGGCAAGGAAG	Sequencing of the STER_0406 locus with #53 (622 pb) [58°C]
**53**	CGCCTTCCCAAGCTTTTTA	Used with #52
**54**	CACGCGATACAGATATTAAGAAGG	Sequencing of the STER_0498/0499 loci with #55 (802 pb) [58°C]
**55**	CGCTACCACAAAAAGCACCT	Used with #54
**56**	AGATGAACTGGATGGGCTTG	Sequencing of the STER_0498/0499 loci with #57 (837 pb) [58°C]
**57**	GCAGGATTTTTCGCTGAGAC	Used with #56
**58**	TGTAGTCTTGCAGGCTAAGGAA	Sequencing of the STER_0498/0499 loci with #59 (818 pb) [58°C]
**59**	CACCAGGAGTTGCCAAAAA	Used with #58
**60**	TGCCCCTAGCTTTGATGAAT	Sequencing of the STER_0498/0499 loci with #61 (1011 pb) [58°C]
**61**	CCAGTGATACCAACGATTGAAA	Used with #60
**62**	TGTGACACCCCAATAAAAATTA	Sequencing of the STER_0572 locus with #63 (631 pb) [58°C]
**63**	GGTGAAATATAAACCACGGGAAG	Used with #62
**64**	TCTTTCACAGGATCAGGTTGC	Sequencing of the STER_0572 locus with #65 (876 pb) [58°C]
**65**	TTGTGACTGAGTCGAAAATGAGA	Used with #64
**66**	CCCAGCTAATTTCGGTTTGA	Sequencing of the STER_0582/0583 loci with #67 (929 pb) [58°C]
**67**	AAATCGTACAAGATACTGATTATTCG	Used with #66
**68**	CTACTCCCAATTAGTAGAAAGTTTCAG	Sequencing of the STER_0582/0583 loci with #69 (623 pb) [58°C]
**69**	CCCTCCTTGAATGGCAGATA	Used with #68
**70**	ATCCCATCAAAGGCTTCGTA	Sequencing of the STER_0582/0583 loci with #71 (916 pb) [58°C]
**71**	TTTTGACTTCAAATATTTACGTTACCT	Used with #70
**72**	GGCAAAGAGACCTGTTCAGTG	Sequencing of the STER_0711/0712 loci with #73 (1089 pb) [58°C]
**73**	TTCTTCTGAGATAAAGCCATCAA	Used with #72
**74**	CCCTCTTTTTAGAAATGTTAGACCA	Sequencing of the STER_0711/0712 loci with #75 (693 pb) [58°C]
**75**	GCTCTAAGATTTGAGGTGAAGGTT	Used with #74
**76**	CGCAGCCCTCTTTATCAATG	Sequencing of the STER_0758 locus with #77 (738 pb) [58°C]
**77**	AACAAGAGAAAAGGCAAGAGC	Used with #76
**78**	GGTATTGCAAATCTTCAAAGTGA	Sequencing of the STER_0807 locus with #79 (794 pb) [58°C]
**79**	TTTGATTCAGTTGCTTTAAGTGG	Used with #78
**80**	AATCACTTTCTGGGAAGTCCA	Sequencing of the STER_0811 locus with #81 (541 pb) [58°C]
**81**	CATTGGCATAATCCATAGTTTCAA	Used with #80
**82**	CCAACAGCTTTAACCGATTGAC	Sequencing of the STER_0901 locus with #83 (780 pb) [58°C]
**83**	CACCGATTTCTGAACGGAAG	Used with #82
**84**	AATGAATGAAGCCGCAAAAC	Sequencing of the STER_0901 locus with #85 (941 pb) [58°C]
**85**	TTTTTGGGGTCAGTACAAAGG	Used with #84
**86**	GAAAATGTCAAGATGCCATATCT	Sequencing of the STER_0949 locus with #87 (699 pb) [58°C]
**87**	CCCATCTCCGATAACGACAA	Used with #86
**88**	AGGCTATCGGAATTGTCGAA	Sequencing of the STER_0949 locus with #89 (958 pb) [58°C]
**89**	GCCCTTTCGACTGTAATCATTT	Used with #88
**90**	ACACCGCTTCCAGAAAGTGT	Sequencing of the STER_0972 locus with #91 (924 pb) [58°C]
**91**	GCCTACCAAGGCGTGTTTT	Used with #90
**92**	CATGCTCGCAACTCATATTTAGTA	Sequencing of the STER_1045/1046/1047/1048 loci with #93 (717 pb) [58°C]

**93**	ACAAAAGATCCGAGCGTGTC	Used with #92
**94**	TTTTCTCGTCAGTTGGTCTCC	Sequencing of the STER_1045/1046/1047/1048 loci with #95 (842 pb) [58°C]
**95**	CATCCCCACTTCCAACTCAC	Used with #94
**96**	TGGAAGATGTAGGCAAGAAGC	Sequencing of the STER_1045/1046/1047/1048 loci with #97 (828 pb) [58°C]
**97**	CAGTAACTAAAGCTAGGACAGTTCCA	Used with #96
**98**	CTGTCTGGTGCTCTTTGTCC	Sequencing of the STER_1045/1046/1047/1048 loci with #99 (777 pb) [58°C]
**99**	CCACCGCCTTCGAGTACTAA	Used with #98
**100**	TCAATCAGTGAGTGCTTTTCG	Sequencing of the STER_1073/1074 loci with #101 (1015 pb) [58°C]
**101**	TGGGAATACGATAACTCCTCCT	Used with #100
**102**	GGGCAACCGTATTTTTAGGG	Sequencing of the STER_1073/1074 loci with #103 (1010 pb) [58°C]
**103**	GCTCTACGCGTCTAAGCAGGT	Used with #102
**104**	GTCTGGCCGAAAAAGTGGTA	Sequencing of the STER_1196/1197 loci with #105 (879 pb) [58°C]
**105**	CAAGGTGATTAAAACCTTTTCCA	Used with #104
**106**	TTGGACCCCTTCACTTATGC	Sequencing of the STER_1196/1197 loci with #107 (732 pb) [58°C]
**107**	TTGGATTGTCCAACTTGCTTC	Used with #106
**108**	TATGCGTGCATCTGGGACTA	Sequencing of the STER_1196/1197 loci with #109 (1406 pb) [58°C]
**109**	TGTTTGTGAGTCTTACTCAAGGCTA	Used with #108
**110**	GGACGACTTGGTTGGCATAG	Sequencing of the STER_1319 locus with #111 (965 pb) [58°C]
**111**	CAAGATTTCTCAATCCTGCTACTTT	Used with #110
**112**	GAGGATGGAAATGTGAAAGCA	Sequencing of the STER_1356/1357 loci with #113 (696 pb) [58°C]
**113**	AGTTCAAGCGGAAAACTCAA	Used with #112
**114**	GGTATTCAATATGATGACAATTTCAGA	Sequencing of the STER_1356/1357 loci with #115 (855 pb) [58°C]
**115**	CCTCAGAATAATTAGCTACTGAAAATC	Used with #114
**116**	TTTTAGGAGGGGAACCGACT	Sequencing of the STER_1356/1357 loci with #117 (1012 pb) [58°C]
**117**	AGGAATATCAAAAACTGAGCCACAT	Used with #116
**118**	TCCGATTTCATCAGCGATAG	Sequencing of the STER_1371 locus with #119 (933 pb) [58°C]
**119**	TCACGACGTTTGTTAGTGTTCA	Used with #118
**120**	CGCTATTGGTATGGGAGCTG	Sequencing of the STER_1371 locus with #121 (936 pb) [58°C]
**121**	GATTGACAACGCTCGGTCTT	Used with #120
**122**	GGACGTTAGCGAACTGCTTT	Sequencing of the STER_1453 locus with #123 (822 pb) [58°C]
**123**	AGCCACTAGATCCCCAAGGT	Used with #122
**124**	TTACGGCTGGATTGAAGGTT	Sequencing of the STER_1453 locus with #125 (1044 pb) [58°C]
**125**	CAGCAACAAGGAAAGCCATC	Used with #124
**126**	TCTTGGATGAGGTGGGAAAA	Sequencing of the STER_1477 locus with #127 (873 pb) [58°C]
**127**	TCTCCTTCAATTAAGAAGTGACCT	Used with #126
**128**	AAAAAGCAGATTTGCGTCTAGT	Sequencing of the STER_1477 locus with #129 (1080 pb) [58°C]
**129**	CCTTCTCTTCTGGCAAATACAAA	Used with #128
**130**	CCTTGGAATTTTGAGGACGTT	Sequencing of the STER_1477 locus with #131 (1076 pb) [58°C]
**131**	TCTCCGGTATACATATCTTTTCCATT	Used with #130
**132**	CGCACTTCAAAATGATCGAC	Sequencing of the STER_1477 locus with #133 (1067 pb) [58°C]
**133**	TTTTCTTAGCACCTTTTTCAATTGTC	Used with #132
**134**	GGGGGTATGCTGGAATTTCT	Sequencing of the STER_1477 locus with #135 (889 pb) [58°C]
**135**	CAATACAATGTCAGTTGTTTCCA	Used with #134
**136**	TCTTATCCCTTGTCATCGTGTTT	Sequencing of the STER_1485 locus with #137 (569 pb) [58°C]
**137**	AGCGGCAACTGTTTCTATCTT	Used with #136
**138**	CTCGGGTGTACAATCGGTCT	Sequencing of the STER_1494 locus with #139 (1087 pb) [58°C]
**139**	AACCAGTTTGTCCGAAGCAC	Used with #138
**140**	AAAAGCTTGAAGCATGGTCAC	Sequencing of the STER_1693 locus with #141 (855 pb) [58°C]
**141**	TGCAAGTAAATAAGTCGATTTGAA	Used with #140
**142**	TGCCTCGCTACTCGACTTTT	Sequencing of the STER_1693 locus with #143 (648 pb) [58°C]
**143**	ACCGTAGCTTATGCGTGTCA	Used with #143
**144**	AAATTCATTCGGTGGTGGAG	Sequencing of the STER_1726 locus with #145 (446 pb) [58°C]
**145**	CTCAGATGAGGGCATTATTTTTAATC	Used with #144
**146**	GGTTTGTTAAGTAGTCAGTTTGATGC	Sequencing of the STER_1743 locus with #147 (593 pb) [58°C]
**147**	CAGCGAAGACGATATTGCAG	Used with #146
**148**	CATGGTGGGAAATGACTTGA	Sequencing of the STER_1814 locus with #149 (1111 pb) [58°C]
**149**	GCTTGCGTTTTAAATTCTTCCA	Used with #148
**150**	GCTTTAGCACAAACGTGGAA	Sequencing of the STER_1824 locus with #151 (941 pb) [58°C]
**151**	ACCAATGATCCCACCAGAAA	Used with #150
**152**	CCAGCTAAATGGCGTACCAA	Sequencing of the STER_1859 locus with #153 (1031 pb) [58°C]
**153**	CAACGATAGCTGCTTGTTCAG	Used with #152
**154**	GCTGGGAATGTCCCATCTTA	Sequencing of the STER_1859 locus with #155 (976 pb) [58°C]
**155**	GCGATAATCACATCTGGTTGG	Used with #154
**156**	CAAACACTTGGCAGGACGTA	Sequencing of the STER_1904/1905/1906 loci with #157 (945 pb) [58°C]
**157**	TGCAGCTTCAACTGCTTGTT	Used with #156
**158**	AGAAGGAAACAAATTCGCTGA	Sequencing of the STER_1904/1905/1906 loci with #159 (604 pb) [58°C]
**159**	TTGTTGTTACGACCAGCTTTG	Used with #158
**160**	CGTGTAGGACGTTACACTGGTT	Sequencing of the STER_1904/1905/1906 loci with #161 (850 pb) [58°C]
**161**	CGTCCAACTGGTGCTTTACC	Used with #160
**162**	GGTAACGCTCTTCCACTTGC	Sequencing of the STER_1904/1905/1906 loci with #163 (557 pb) [58°C]
**163**	CGTCCCATTTTGTAGTTTTCTCC	Used with #162
**164**	TGGAAGATAAAGAGACAAAACAAAGA	Sequencing of the STER_1951 locus with #165 (865 pb) [58°C]

**165**	CGCAAAGGATTTTCTTCCAA	Used with #164
**166**	CCAGAGGTCCAAAAACCTGT	Sequencing of the STER_1951 locus with #167 (847 pb) [58°C]
**167**	TGGCTTTGAAGGTATCTGCTG	Used with #166
**168**	CCAATATGGTACGTGGTCTGG	Sequencing of the STER_1951 locus with #169 (682 pb) [58°C]
**169**	GATCACTCGCAGGTTCATCA	Used with #168
**170**	TTCGCTTTTGAAGTGTGACG	Sequencing of the STER_1952 locus with #171 (583 pb) [58°C]
**171**	TACCGTTGATACGGACGTTG	Used with #170
**172**	GGTGGCACCTTGATTTCCTA	Sequencing of the STER_1952 locus with #173 (973 pb) [58°C]
**173**	TCCACGAAACTTTTTCTTGCT	Used with #172
**174**	TCTCCTAAGTGTTTCACTAGCTTCAA	Sequencing of the STER_1968 locus with #175 (1040 pb) [58°C]
**175**	GGCTAATTTGAGCTGGCTTTT	Used with #174
**176**	TTGGACGAACTAAAATAGTGACG	Sequencing of the STER_B1 locus with #177 (603 pb) [58°C]
**177**	AGACGTCTCTTTTCGTAATTTTTAAT	Used with #176

The numbers in parentheses and square brackets correspond to expected amplicons sizes and PCR annealing temperatures, respectively. †: A touchdown PCR was applied for the first 20 cycles with a 1°C decrease per cycle from 60°C to 40°C followed by a constant 42°C annealing temperature for the last 30 cycles. *: Sequences in bold and italics correspond to loxP sites.

Table S2. Inserts from activated R-IVET clones identified at T0 in the different gut models

**Insert**	**Locus STER_[a] (STER_RS0)[b]**	**Gene description**	**Location and direction compared to the gene[c]**	**Position of the insert on LMD-9 genome (nt)**	**TIM model**	**Caco-2 TC7**	**Batch cultures of human fecal microbiota**
**1**	B2 (9835)	Plasmid rolling circle replication initiator protein	Promoter/sense	2082-2433		LM17/DMEM	
**2**	0060 (0310)	Phosphoribosylaminoimidazole carboxylase ATPase subunit	Inside/sense	48325-49378			
**3**	0071 (0360)	Hypothetical protein	Promoter/sense	58621-59672			
**4**	0095 (0480)	*polC*: DNA polymerase III catalytic subunit, PolC	Inside/sense	75226-76208		DMEM	
**5**	0096 (0485)	PepS aminopeptidase. Metallo peptidase. MEROPS family M29	Promoter sense	77288-77475		DMEM	
**6**	0113 (0565)	D-alanyl-D-alanine carboxypeptidase, penicillin-binding protein	Promoter/sense	93170-92322		LM17/DMEM	
**7**	0120 (0595)	Hypothetical protein	Promoter/sense	98597-99644			
**8**	0124 (0615)	Hypothetical protein	Inside/sense	102285-102381		DMEM	
**9**	0125 (0620)	Hypothetical protein	Promoter/sense	102382-102740		LM17	
**10**	0148 (0715)	Transposase	Promoter/antisense	122595-122799			
**11**	0206 (1015)	Crp-like transcriptional regulator	Promoter/sense	175028-174569		LM17/DMEM	
**12**	0207 (1020)	Permease of the major facilitator superfamily	Promoter/sense	174822-175620		LM17	
**13**	0207 (1020)	Permease of the major facilitator superfamily	Inside/sense	175029-175686		DMEM	
**14**	0236 (1155)	Auxin efflux carrier (AEC) family permease	Promoter/sense	200160-200652		LM17	
**15**	0245 (1200)	CDP-diglyceride synthetase	Inside/antisense	208815-208140		LM17	
**16**	0384 (1865)	*rbfA*: ribosome-binding factor A	Promoter/sense	336578-337533		LM17/DMEM	
**17**	0468 (2300)	N-formylmethionyl-tRNA deformylase	Promoter/sense	414741-415335		LM17/DMEM	
**18**	0509 (1030)	Glycopeptide antibiotics resistance protein	Promoter/sense	457780-457906		LM17	
**19**	0531 (2605)	Ammonia permease	Promoter/sense	477410-477541		LM17	
**20**	0537 (2635)	N-acetylglucosamine 6-phosphate deacetylase	Promoter/sense	481688-482351		LM17	
**21**	0573 (2825)	Predicted protein	Promoter/sense	517470-518024			
**22**	0583 (2870)	Response regulator of the LytR/AlgR family	Promoter/sense	531843-530990			
**23**	0611 (3000)	Glycosyl transferase, family 1	Inside/sense	555151-556148			
**24**	0612 (3005)	Hypothetical protein	Promoter/sense	556224-556595		DMEM	

**25**	0623 (3055)	Dihydrofolate reductase	Promoter/sense	566220-566539	
**26**	0623 (3055)	Dihydrofolate reductase	Promoter/sense	566220-566311	LM17
**27**	0660 (3255)	Methenyltetrahydrofolate cyclohydrolase	Promoter/sense	598595-598901	LM17
**28**	0711 (3500)	CRISPR-associated protein, Cas2 family	Promoter/sense	647294-647916	
**29**	0744 (3650)	Uncharacterized conserved protein	Promoter/sense	675683-675866	
**30**	0747 (3665)	Type I site-specific restriction-modification system, R (restriction) subunit or related helicase	Inside/sense	679498-680421	
**31**	0761 (3735)	*holA*: DNA polymerase III subunit delta	Promoter/sense	694068-694911	
**32**	0813 (4000)	Uncharacterized conserved phage related protein	Promoter/sense	747866-747426	
**33**	0830 (4075)	Hypothetical protein	Promoter/sense	755652-756572	
**34**	0872 (4310)	Enzyme related to GTP cyclohydrolase I	Promoter/sense	802930-803563	LM17
**35**	0885 (4380)	Cystathionine beta-synthase (acetylserine-dependent)	Promoter/sense	817241-817531	
**36**	0948 (4690)	Ferrochelatase	Promoter/sense	877316-878238	LM17/DMEM
**37**	0949 (4595)	Threonine dehydrogenase or related Zn-dependent dehydrogenase	Promoter/sense	879316-879396	LM17
**38**	0950/0951 (-/4700)	Hypothetical protein/pseudogene	Promoter/sense	879949-880988	LM17
**39**	0981 (4840)	*pyrE*: Orotate phosphoribosyltransferase	Promoter/sense	905098-905735	DMEM
**40**	1012 (4990)	Lysyl aminopeptidase. Metallo peptidase. MEROPS family M01	Inside/sense	934009-935056	
**41**	1013 (4995)	*xerS*: Site-specific tyrosine recombinase XerS	Promoter/sense	936928-935885	LM17/DMEM
**42**	1043 (5175)	Pseudogene	Promoter/sense	967939-968905	LM17
**43**	1102 (5460)	*prfB*: peptide chain release factor 2	Promoter/sense	1019356-1018918	
**44**	1166 (5755)	Putative translation factor (SUA5)	Promoter/sense	1078437-1078262	LM17/DMEM
**45**	1197 (5905)	Cell division membrane protein	Promoter/sense	1106940-1106003	
**46**	1238 (6110)	Uridine kinase	Promoter/sense	1144304-1143294	LM17
**47**	1280 (6320)	*urvC*: excinuclease ABC subunit C	Inside/sense	1190055-1189837	LM17/DMEM
**48**	1293 (6375)	ABC-type multidrug transport system, ATPase component	Promoter/antisense	1201333-1202291	
**49**	1298 (-)	Hypothetical protein	Promoter/sense	1206867-1207715	
**50**	1320 (6520)	Predicted ring-cleavage extradiol dioxygenase	Inside/antisense	1228239-1228286	DMEM
**51**	1356 (6690)	Radical SAM superfamily enzyme	Inside/sense	1260895-1260614	LM17/DMEM
**52**	1400 (6885)	Predicted hydrolase of the HAD superfamily	Promoter/sense	1310880-1309989	
**53**	1406 (6915)	ABC-type dipeptide/oligopeptide/nickel transport system, ATPase component	Inside/sense	1314940-1314560	LM17/DMEM
**54**	1408 (6925)	ABC-type dipeptide/oligopeptide/nickel transport system, permease component	Inside/sense	1317075/1316209	
**55**	1453 (7150)	Aminotransferase	Promoter/sense	1361583-1361200	DMEM

**56**	1469 (7225)	Uncharacterized protein, possibly involved in aromatic compounds catabolism	Promoter/sense	1374989-1374939	LM17/DMEM
**57**	1518 (7470)	SSU ribosomal protein S5P alanine acetyltransferase	Promoter/sense	1422905-1422091	LM17
**58**	1522 (7490)	1-acyl-sn-glycerol-3-phosphate acyltransferase	Promoter/sense	1428562-1427572	
**59**	1539 (7565)	ABC-type amino acid transport/signal transduction system, periplasmic component/domain	Promoter/sense	1443950-1443474	DMEM
**60**	1579 (7760)	Hypothetical protein	Inside/antisense	1479868-1480392	LM17/DMEM
**61**	1585 (7790)	Predicted GTPase	Inside/antisense	1484613-1485240	
**62**	1586 (7795)	Predicted hydrolase of the HAD superfamily	Promoter/sense	1486730-1486347	LM17
**63**	1591 (7815)	*gatA*: aspartyl/glutamyl-tRNA(Asn/Gln) amidotransferase subunit A	Inside/sense	1490454-1490313	DMEM
**64**	1597 (7840)	ABC-type metal ion transport system, periplasmic component/surface antigen	Promoter/sense	1495479-1495063	
**65**	1643 (8060)	Hypothetical protein	Promoter/sense	1535445-1535290	LM17
**66**	1664 (8140)	Superfamily II DNA and RNA helicase	Promoter/sense	1554241-1553929	LM17
**67**	1684 (8235)	Response regulator of the LytR/AlgR family	Promoter/sense	1576456-1576201	LM17/DMEM
**68**	1736 (8480)	DNA polymerase I	Promoter/sense	1625236-1624238	LM17
**69**	1774 (8680)	Mg-dependent DNase	Promoter/sense	1659581-1659384	LM17/DMEM
**70**	1846 (9020)	Membrane carboxypeptidase (penicillin-binding protein)	Promoter/sense	1723432-1723281	DMEM
**71**	1862 (9110)	Pseudogene	Promoter/sense	1743723-1742676	LM17
**72**	1866 (9140)	Hypothetical protein	Promoter/sense	1745938-1745578	
**73**	1895 (9285)	LSU ribosomal protein L5P	Promoter/sense	1763621-1763389	
**74**	1912/1911 (9365/9370)	Protein-tyrosine-phosphatase/Uncharacterized protein conserved in bacteria	Promoter/sense	1773889-1772913	
**75**	P1: 0061 (0315)	Pseudogene	Inside/antisense	50147-50034	LM17/DMEM
	P2: -	No gene identified	-	428255-428540	
**76**	P1: 0107 (0535)	Predicted membrane protein	Inside/antisense	85074-85135	LM17/DMEM
	P2: 1980 (9680)	Hypothetical protein	Promoter/sense	1834023-1833850	
	P3: 0338 (-)	Hypothetical protein	Promoter/sense	292729-292793	
**77**	P1: 0157 (0760)	Uncharacterized conserved protein	Inside/sense	129818-129958	LM17
	P2: 0633 (3105)	DNA topoisomerase IV subunit B	Inside/sense	575858-576757	
**78**	P1: 0220 (1070)	Cysteine desulfurase	Inside/sense	186671-187317	LM17/DMEM
	P2: 1772 (8670)	*ksgA*: dimethyladenosine transferase	Promoter/sense	1658295-1657961	
**79**	P1: 0267 (1295)	Pseudogene	Inside/antisense	227033-227182	LM17/DMEM
	P2: B2 (9835)	Plasmid rolling circle replication initiator protein	Inside/antisense	2435-2300	
**80**	P1: 0307 (1490)	*scpA*: segregation and condensation protein A	Inside/sense	264475-264593	DMEM

	P2: 1111 (5495)	Predicted hydrolase of the HAD superfamily	Promoter/sense	1025666-1026525	
**81**	P1: 0311 (1510)	Trk-type K+ transport system, membrane component	Promoter/antisense	268092-268381	LM17
	P2: 0198 (0970)	5'-nucleotidase/2',3'-cyclic phosphodiesterase or related esterase	Inside/antisense	164148-164035	
	P3: 0662 (3265)	Cell division protein FtsI/penicillin-binding protein 2	Promoter/sense	600539-601100	
**82**	P1: 0351 (1710)	Trk-type K+ transport system, membrane component	Inside/antisense	307122-307554	LM17
	P2: 0839 (4130)	Pseudogene	Inside/antisense	768120-767545	
**83**	P1: 0388 (1890)	Membrane protein involved in polysaccharide transport	Inside/sense	342566-342669	LM17/DMEM
	P2: 0744 (3650)	Uncharacterized conserved protein	Promoter/sense	675681-675865	
**84**	P1: 0528 (2590)	Regulator of signaling phosphorelay	Promoter/antisense	475645-474908	LM17
	P2: -	No gene identified	-	832730-832547	
**85**	P1: 0694 (3415)	Malate dehydrogenase (NAD)	Inside/antisense	630957-630878	DMEM
	P2: 1123 (5555)	UDP-N-acetylglucosamine 1-carboxyvinyltransferase	Promoter/sense	1037911-1036955	
**86**	P1: 0751 (3685)	Hypothetical protein	Inside/antisense	684811-184699	LM17/DMEM
	P2: 1513 (7445)	NAD-dependent DNA ligase	Inside/sense	1418027-1417943	
	P3: 1524 (7500)	Hypothetical protein	Promoter/sense	1428673-1428787	
**87**	P1: 0761 (3735)	*holA*: DNA polymerase III, delta subunit	Promoter/sense	694511-694643	LM17
	P2: 0699 (3440)	Tellurite resistance protein or related permease	Inside/antisense	636263-636379	
**88**	P1: 0777 (3810)	Cell division protein FtsZ	Inside/antisense	710969-710860	DMEM
	P2: 1454 (7155)	ABC-type amino acid transport/signal transduction system, periplasmic component/domain	Promoter/antisense	1362197-1363049	
**89**	P1: 1103 (-)	Hypothetical protein	Promoter/sense	1019044-1018908	LM17
	P2: 0375 (1820)	Xanthine/uracil/vitamin C permease	Inside/sense	329323-329405	
	P3: 1861 (9105)	Pseudogene	Inside/sense	1742078-1741907	
**90**	P1: 1134 (5600)	Hypothetical protein	Inside/antisense	1047111-1047304	
	P2: 0289 (1400)	*greA*: transcription elongation factor GreA	Promoter/sense	251808-252550	
**91**	P1: 1139 (5625)	GAF domain-containing protein	Promoter/sense	1054731-1054392	LM17/DMEM
	P2: 1715 (8380)	Translation elongation factor P (EF-P)	Inside/sense	1606151-1606065	
**92**	P1: 1379 (6790)	Glutamate-cysteine ligase	Inside/antisense	1287692-1287449	LM17/DMEM
	P2: 0336 (1635)	NUDIX family hydrolase	Promoter/antisense	289592-288847	
**93**	P1: 1400 (6885)	Predicted hydrolase of the HAD superfamily	Inside/sense	1310087/1309642	LM17/DMEM
	P2: 0574 (2830)	Uncharacterized conserved phage related protein	Inside/sense	518690-519174	
**94**	P1: 1413 (6950)	Transposase	Inside/antisense	1324280-1324120	
	P2: 0252 (1225)	*groES*: co-chaperonin GroES (HSP10)	Promoter/sense	215497-215643	

	P3: 0252 (1225)	*groES*: co-chaperonin GroES (HSP10)	Promoter/sense	215675-215798	
**95**	P1: 1461 (7190)	ABC-type amino acid transport system, periplasmic and permease components	Inside/antisense	1369441-1369027	
	P2: 1710 (8355)	Sucrose PTS, EIIBCA	Inside/sense	1600881-1600629	
	P3: 1489 (7320)	Phosphoserine aminotransferase	Promoter/sense	1395106-1394818	
**96**	P1: 1534 (7550)	UDP-N-acetylmuramoyl-tripeptide--D-alanyl-D-alanine ligase	Promoter/sense	1441347-1440845	
	P2: 0862 (4250)	Uncharacterized protein conserved in bacteria	Inside/sense	796404-796597	
	P3: 0864 (4260)	*asnC*: asparaginyl-tRNA synthetase	Inside/sense	798722-799012	
**97**	P1: 1676 (8195)	Glutamate 5-kinase	Promoter/sense	1564089-1563756	LM17/DMEM
	P2: 1797 (8780)	*rplA*: LSU ribosomal protein L1P	Inside/antisense	1681062-16811278	
**98**	P1: 1682 (8225)	DNA helicase/exodeoxyribonuclease V, subunit B	Promoter/sense	1574798-1574554	LM17/DMEM
	P2: 0389 (1895)	Pseudogene	Promoter/antisense	343134-343016	
**99**	P1: 1684 (8235)	Response regulator of the LytR/AlgR family	Promoter/sense	1576456-1576301	
	P2: 0383 (1860)	*infB*: bacterial translation initiation factor 2 (bIF-2)	Inside/antisense	336251-335993	
**100**	P1: 1689 (8255)	Uncharacterized protein conserved in bacteria (Cof family protein)	Inside/sense	1578827-1578740	LM17/DMEM
	P2: 1000 (4930)	*truB*: tRNA pseudouridine synthase B	Promoter/sense	922786-922977	
**101**	P1: 1690 (8260)	Asparaginase	Inside/antisense	1581011-1580885	LM17
	P2: 0165 (0800)	*truA*: Pseudouridine synthase A	Inside/antisense	139087-138850	
	P3: -	No gene identified	-	1270201-1270550	
**102**	P1: 1709 (8350)	Fructokinase	Inside/sense	1599180-1599064	DMEM
	P2: 1184 (5845)	*xseA*: exodeoxyribonuclease VII large subunit	Promoter/sense	1094776-1093928	
**103**	P1: 1746 (8530)	Acetyltransferase	Inside/antisense	1635078-1635212	
	P2: 1180 (5825)	Transcriptional regulator, ArgR family	Promoter/sense	1091139-1090473	
**104**	P1: 1827 (8925)	Glutamyl aminopeptidase. Metallo peptidase. MEROPS family M42	Promoter/sense	1702403-1702595	DMEM
	P2: 0760 (3730)	Phosphoenolpyruvate carboxylase	Inside/antisense	692925-692835	
**105**	P1: 1857 (9070)	L-threonine synthase	Inside/sense	1736178-1735869	
	P2: 0761 (3735)	*holA*: DNA polymerase III, delta subunit	Promoter/sense	694250-694640	
**106**	P1: 1889 (8255)	*rpmD*: 50S ribosomal protein L30	Inside/antisense	1760360-1760519	LM17/DMEM
	P2: 1555 (7645)	Chorismate mutase	Promoter/sense	1458978-1458361	
**107**	P1: 1946 (9515)	Hypothetical protein	Promoter/sense	1804618-1804321	LM17/DMEM
	P2: 0126 (0625)	Ketopantoate reductase	Inside/antisense	103934-103277	
**108**	P1: 1969 (9630)	Predicted membrane protein	Inside/antisense	1820741-1820903	

	P2: 1992 (9740)	Inosine-5'-monophosphate dehydrogenase	Promoter/antisense	1845233-1846062			
**109**	P1: 1969 (9630)	Predicted membrane protein	Inside/antisense	1820633-1820859		DMEM	
	P2: 0595 (2930)	Pseudogene	Promoter/antisense	541106-540512			
**110**	P1: -	No gene identified	-	429681-429550		DMEM	
	P2: 0787 (3860)	*rpmE2*: LSU ribosomal protein L31P	Promoter/sense	720571-720098			

([a]): locus annotation dating from 01-28-2014; ([b]): locus annotation dating from 07-30-2015 on NCBI; ([c]): See Fig. 5 for term explanations. P1, P2 and P3: when a single insert has overall or part of its sequence which aligns with different (two or three) regions of LMD-9 genome.

Table S3. Allele variability of R-IVET activated genes identified in TIM-1 and fecal batch cultures

		Gene											Protein												
	***S. thermophilus* classes**[c]	**6**	**6**	**6**	**5**	**4**	**4**	**3**	**2**	**2**	**2**	**1**	**6**	**6**	**6**	**5**	**4**	**4**	**3**	**2**	**2**	**2**	**1**		
	**Resistance level to GI stresses**[d]	**High level**	**High level**	**High level**	**High level**	**Intermediate level**	**Intermediate level**	**Intermediate level**	**Low level**	**Low level**	**Low level**	**High level**	**High level**	**High level**	**High level**	**High level**	**Intermediate level**	**Intermediate level**	**Intermediate level**	**Low level**	**Low level**	**Low level**	**High level**	**Signature High Level**[e]	**Signature Low Level**[f]
**Locus STER_**[a] **(STER_RS)**[b]	**Gene description**	**LMD-9**[g]	PB18O	PB302	EBLST20	LMG18311	CNRZ160	PB385	EBL308	ST14	CNRZ21	EBL385	**LMD-9**[g]	PB18O	PB302	EBLST20	LMG18311	CNRZ160	PB385	EBL308	ST14	CNRZ21	EBL385		
**0208 (01025)**	30S ribosomal protein S15	1	1	1	1	2	2	1	3	4	4	1	a	a	a	a	a	a	a	a	a	a	a	**a**	**a**
**0303 (01470)**	Non-canonical purine NTP pyrophosphatase	1	2	2	2	3	4	2	5	6	1	2	a	b	b	b	c	c	b	d	e	a	b	**b**	**a/d/e/**
**0399 (01945)**	Branched-chain amino acid ABC-type transport system, permease component	1	2	2	2	3	4	2	5	6	3	7	a	a	a	a	a	a	a	a	a	a	b	**a**	**a**
**0498 (02445)**	Predicted amydohydrolase	1	2	2	2	3	ND	2	ND	4	3	5	a	b	b	b	c	ND	b	ND	d	c	b	**b**	**c/d**
**0499 (02450)**	Transaminase	1	1	1	1	2	3	1	4	5	2	6	a	a	a	a	b	a	a	c	d	b	e	**a**	**b/c/d**
**0572 (02820)**	ABC permease transporter	1	1	1	1	-	-	1	-	-	-	2	a	a	a	a	-	-	a	-	-	-	b	**a**	**-**
**0901 (04465)**	LysR family transcriptional regulator	1	1	1	1	2	3	1	4	3	2	5	a	a	a	a	b	a	a	b	a	b	c	**a**	**b**
**0949 (04695)**	Zn-dependant alcohol dehydrogenase	1	1	1	1	2	3	1	4	3	2	3	a	a	a	a	b	a	a	c	a	b	a	**a**	**a/b/c**
**0972 (04800)**	CRISPR-associated endoribonuclease Cas6	1	1	1	1	2	-	1	3	4	2	5	a	a	a	a	b	-	a	c	c	b	d	**a**	**c**
**1046 (5190)**	Hypothetical protein	1	1	1	1	2	3	1	4	5	2	4	a	a	a	a	b*	c**	a	d**	b*	b*	d**	**a**	**b***
**1047 (05195)**	Predicted unusual protein kinase	1	2	2	2	3	4	2	5	6	3	ND	a	b	b	b	c	d	b	e	f	c	ND	**b**	**c/e/f**
**1048 (05200)**	Hypothetical protein (phasin protein superfamily)	1	1	1	1	2	3	1	4	5	2	ND	a	a	a	a	b	b	a	c	d	b	ND	**a**	**b/c/d**
**1319 (06515)**	Hypothetical protein (nitroreductase-like protein family)	1	2	2	2	3	4	5	6	7	8	9	a	b	b	b	c	d	b	e	f	g	e	**b**	**e/f/g**
**1356 (06690)**	KxxxW cyclic peptide radical SAM maturase	1	1	1	1	-	-	1	-	-	-	1	a	a	a	a	-	-	a	-	-	-	a	**a**	**-**
**1357 (10575)**	Hypothetical protein	1	1	1	1	-	-	1	-	-	-	1	a	a	a	a	-	-	a	-	-	-	a	**a**	**-**
**1693 (08275)**	Trancriptional regulator (helix-turn-helix XRE-family like protein)	1	1	1	1	-	-	1	-	-	-	-	a	a	a	a	-	-	a	-	-	-	-	**a**	**-**

**1814 (08865)**	2,3,4,5-tetrahydropyridine-2,6-dicarboxylate N-acetyltransferase	1	1	1	1	2	1	1	3	1	2	4	a	a	a	a	a	a	a	a	a	a	a	**a**	**a**
**1824 (08910)**	EMAP domain-containing protein	1	1	1	1	2	3	1	4	5	6	7	a	a	a	a	a	a	a	a	b	a	a	**a**	**a**
**1726 (08440)**	30S ribosomal protein S18	1	1	1	1	1	1	1	1	1	1	1	a	a	a	a	a	a	a	a	a	a	a	**a**	**a**
**1905 (09335)**	LSU ribosomal protein L23P	1	1	1	1	1	1	1	1	2	1	1	a	a	a	a	a	a	a	a	a	a	a	**a**	**a**
**1906 (09340)**	50S ribosomal protein L4	1	1	1	1	2	2	1	1	3	2	1	a	a	a	a	a	a	a	a	b	a	a	**a**	**a**
**1951 (09535)[h]**	Histidyl-tRNA synthetase	1	1	1	1	2	3	1	4	2	5	6	a	a	a	a	b	c	a	d	b	b	a	**a**	**b**
**r0184 (00895)[i]**	rRNA-5S ribosomal RNA	1	1	1	1	1	1	1	1	1	1	1													
**t0185 (00900)[i]**	tRNA-Asn	1	1	1	1	1	1	1	1	1	1	1													

([a]): locus annotation from 01-28-2014; ([b]): locus annotation from 07-30-2015 on NCBI; ([c]): according to Junjua *et al.*, 2016; ([d]): GIT stresses include acid pH, bile salts and oxidative stresses; ([e]): type of proteins found in strains exhibiting a high level of resistance against acid stress; ([f]): type of proteins found in strains exhibiting a low level of resistance against acid stress ([g]): LMD-9 used as a reference strain for comparison; ([h]): gene identified in a previous study (Uriot *et al.*, 2016); ([i]): no protein for these genes. (*): stop codon after 20 amino acids; (**): no stop codon identified. (-): no PCR amplicon; ND: PCR amplicons but with no analysable sequence.

Table S4. Allele variability of R-IVET activated genes identified in Caco-2 TC7 cells

		**Gene**								**Protein**							
	***S. thermophilus* classes**[c]	**6**	**6**	**5**	**5**	**4**	**4**	**2**	**1**	**6**	**6**	**5**	**5**	**4**	**4**	**2**	**1**
	**Adhesion capacity to HT29-MTX intestinal cells**	**High adhesion level**	**Intermediate adhesion level**	**High adhesion level**	**High adhesion level**	**Low adhesion level**	**High adhesion level**	**Low adhesion level**	**Intermediate adhesion level**	**High adhesion level**	**Intermediate adhesion level**	**High adhesion level**	**High adhesion level**	**Low adhesion level**	**High adhesion level**	**Low adhesion level**	**Intermediate adhesion level**
**Locus STER_**[a] **(STER_RS)**[b]	**Gene description**	**LMD-9**[d]	PB18O	ST88	EBLST20	PB5MJ	LMG18311	CNRZ21	EBL385	**LMD-9**[d]	PB18O	ST88	EBLST20	PB5MJ	LMG18311	CNRZ21	EBL385
**0004 (00020)**	Hypothetical protein	1	1	1	1	1	1	2	-	a	a	a	a	a	a	b	-
**0314 (01525)**	Membrane protein	1	1	1	1	1	2	3	4	a	a	a	a	a	b	b	b
**0406 (01985)**	Competence protein	1	1	1	1	1	2	2	3	a	a	a	a	a	b	b	c
**0582 (02865)**	Predicted signal transduction protein with C-terminal HATPase domain	1	1	1	1	1	2	1	1	a	a	a	a	a	b	a	a
**0583 (02870)**	DNA-binding response regulator	1	1	1	1	1	2	2	1	a	a	a	a	a	b	b	a
**0711 (03500)**	CRISPR-associated protein, Cas2 family	1	2	1	2	1	3	3	ND	a	b	a	b	a	c	c	ND
**0712 (03505)**	Uncharacterized conserved protein	1	ND	1	2	1	3	4	ND	a	ND	a	b	a	c	d	ND
**0758 (03720)**	Membrane protein	1	1	1	1	1	2	3	4	a	a	a	a	a	b	c	d
**0807 (03965)**	Hypothetical protein	1	2	1	2	1	3	4	-	a	b*	a	b*	a	c**	d*	-
**0811 (03990)**	Transposase	1	1	1	1	1	-	-	1	a	a	a	a	a	-	-	a
**1196 (05900)**	IS5 family transposase	1	1	1	1	1	-	-	-	a	a	a	a	a	-	-	-
**1197 (05905)**	Rod shape-determining protein RodA	1	1	1	1	1	2	3	4	a	a	a	a	a	b	c	a
**1371 (06720)**	Galactokinase	1	ND	1	2	1	3	4	5	a	ND	a	b	a	b	b	c
**1453 (07150)**	Glutamine amidotransferase	1	1	1	1	1	2	2	3	a	a	a	a	a	b	b	c
**1477 (07260)**	Type II CRISPR RNA-guided endonuclease Cas9	1	1	1	1	1	-	-	2	a	a	a	a	a	-	-	b
**1485 (07300)**	Arsenate reductase family protein	1	1	1	1	1	1	2	2	a	a	a	a	a	a	b	b
**1494 (07350)**	Spermidine/purescine ABC transporter permease	1	1	1	1	1	2	3	1	a	a	a	a	a	b	c	a
**1743 (08515)**	Branched-chain amino acid transporter	1	1	1	1	1	2	2	3	a	a	a	a	a	b	b	b
**1968 (09625)**	Hypothetical protein	1	1	1	1	1	2	2	1	a	a	a	a	a	b	b	a
**B1 (09830)**	Acid-shock protein	1	-	1	1	1	-	-	-	a	-	a	a	a	-	-	-

([a]): locus annotation dating from 01-28-2014; ([b]): locus annotation dating from 07-30-2015 on NCBI; ([c]): according to Junjua *et al.*, 2016; ([d]): LMD-9 used as a reference strain for comparison. (*): an extra tryptophan at the end of sequence; (**): stop codon after 60 amino acids. (-): no PCR amplicon; ND: No analysable sequence.
